# Distinct Post-Exposure Metabolic States Following Pharmacological Perturbation of Mitochondrial Respiratory Complexes I–V in SH-SY5Y Cells: A Positional ^13^C-NMR Study

**DOI:** 10.3390/ijms27209160

**Published:** 2026-10-15

**Authors:** Hong Li, Ahmet Tarık Baykal

**Affiliations:** 1 Center for Advanced Metabolomics and Proteomics Research, Department of Microbiology, Biochemistry, and Molecular Genetics, New Jersey Medical School, Rutgers University, Newark, NJ 07103, USA; liho2@njms.rutgers.ed; 2 Department of Medical Biochemistry, Faculty of Medicine, Acibadem Mehmet Ali Aydinlar University, Istanbul 34450, Turkey; 3 Department of Biochemistry and Molecular Biology, Graduate School of Health Sciences, Acibadem Mehmet Ali Aydinlar University, Istanbul 34450, Turkey; 4 Acibadem Labmed Clinical Laboratories, Istanbul 34450, Turkey

**Keywords:** ^13^C NMR metabolomics, stable isotope-resolved metabolomics, electron transport chain, pyruvate carboxylase, SH-SY5Y neuroblastoma, mitochondrial neurotoxicity, respiratory-chain pharmacology, stable isotope-resolved metabolomics, positional ^13^C-NMR, pyruvate carboxylase, anaplerosis, inhibitor polypharmacology

## Abstract

Mitochondrial dysfunction is a recurring mechanism in neuronal vulnerability and in the toxicity of several neuroactive compounds, yet lesions at different respiratory-chain nodes are commonly treated as a single bioenergetic defect. Whether pharmacological perturbation at distinct nodes leaves neuronal cells in distinguishable metabolic states has not been examined across all five complexes in one system. Undifferentiated SH-SY5Y human neuroblastoma cells were exposed to five compounds selected as pharmacological perturbagens nominally directed at Complexes I–V: MPTP, 3-nitropropionic acid (3-NP), antimycin A, sodium azide and oligomycin, each titrated to approximately 80% viability (24 h, except 3-NP at 12 h). Inhibitors were then removed, and cells were labelled for 24 h with [U-^13^C_6_]glucose. Positional ^13^C-NMR was combined with pyruvate carboxylase (PC) and pyruvate dehydrogenase (PDH) enzyme-capacity assays in parallel extracts from the same biological replicates (n = 3 per group). The design is a single-factor, six-level layout in which five exposures share one vehicle control; labelling data were log2-transformed and analysed by one-way ANOVA with Dunnett many-to-one contrasts (error df = 12), with Hedges’ g and 95% confidence intervals, and group structure was tested by permutational multivariate analysis of variance (PERMANOVA). PERMANOVA demonstrated a significant overall effect of exposure group on the multivariate labelling matrix (pseudo-F = 18.77, *p* = 0.0001, 9999 permutations), and principal component analysis of the seven complete-case metabolites showed visually distinct group structure (PC1 65.9%, PC2 19.3%; 85.3% cumulative). Oligomycin produced the most distinct positional phenotype, preserving the PDH-associated glutamate C4 readout (107.2% of control, *p* = 0.971) while nearly doubling the PC-associated glutamate C3 readout (197.7%, *p* = 0.004; Hedges’ g = 17.8, 95% CI 5.3–30.2), concordant with PC capacity at approximately 138% and PDH capacity at approximately 60% of control. Azide produced the strongest reduction in glutamate C4 labelling (31.9%, *p* < 0.001; g = −8.3); antimycin A produced the broadest suppression of labelling; 3-NP produced accumulation of ^13^C-labelled succinate that was undetectable in the control. MPTP gave the weakest phenotype, with most of its individual comparisons not surviving the shared-control analysis. Exposure to compounds directed at different respiratory nodes leaves neuronal cells in non-equivalent post-exposure ^13^C-labelling states that positional ^13^C-NMR resolves. Because tracing followed washout, because the compounds are not fully selective and because the enzyme assays report capacity rather than flux, these are compound-defined post-exposure metabolic states and not measurements of complex-specific flux.

## 1. Introduction

Neurons and neuronal-like cells depend heavily on mitochondrial oxidative metabolism, and mitochondrial dysfunction is a recurring mechanism in neuronal vulnerability and in the toxicity of several neuroactive compounds. Mitochondrial electron transport chain (ETC) lesions are nevertheless frequently discussed as a single bioenergetic defect, yet inhibition of Complexes I to V should redistribute carbon differently, because each lesion imposes a distinct constraint on redox balance, on TCA-cycle continuity or on ATP synthesis. Respiration supports cellular metabolism not only through ATP generation but also through the maintenance of electron-acceptor availability and of biosynthetic competence, including aspartate-linked anaplerotic capacity [1,2,3,4,5]. If different pharmacological lesions leave neuronal cells in different metabolic states, then the metabolic vulnerability conferred by a mitochondrial insult is not a single quantity, and understanding how ETC dysfunction alters carbon fate, rather than merely lowering metabolite abundance, requires a node-specific framework [6].

SH-SY5Y cells offer a practical neuronal-like discovery model for this question: they are widely used in studies of mitochondrial stress and retain dynamic mitochondrial control over bioenergetics across both differentiated and undifferentiated states [7,8,9,10]. Undifferentiated SH-SY5Y cells do not fully reproduce the oxidative dependence of mature neurons, but they do permit controlled side-by-side comparison of multiple ETC lesions in a common cellular background. Previous comparative work in this line has been confined either to single toxins [11] or to viability and oxidative-stress endpoints across several Complex I-affecting toxins [12].

Stable isotope-resolved metabolomics (SIRM) with [U-^13^C_6_]glucose yields pathway-associated information that steady-state untargeted metabolomics cannot, because it tracks the positional fate of labelled carbon as that carbon moves through glycolysis, oxidative TCA entry and anaplerotic entry [13,14,15,16,17]. Comparative [U-^13^C_6_]glucose tracing across several complex-specific ETC inhibitors is already established in primary human retinal pigment epithelium, where Zhang and colleagues showed that piericidin (Complex I), antimycin (Complex III) and oligomycin (Complex V) generate distinguishable metabolic signatures by LC-MS-based targeted metabolomics [18]. Two questions remain open. The first is whether the same separation extends to Complex II and Complex IV, the two complexes that study did not address. The second is whether the positional ^13^C information accessible to ^13^C NMR, and specifically the glutamate C3 versus C4 contrast that discriminates PC-route from PDH-route TCA entry [13,14,16,17], supports finer assignment than the mass-isotopomer patterns available to LC-MS.

This study used positional ^13^C labelling to compare glycolytic carbon disposal, oxidative entry into the TCA cycle and PC-associated anaplerotic labelling across all five ETC lesions in undifferentiated SH-SY5Y cells. Because the experiment terminates at a 24 h isotopic labelling endpoint, the resulting isotopologue patterns are interpreted as pathway-associated labelling signatures and not as direct flux measurements [15]. We hypothesised that exposure to inhibitors directed at different respiratory complexes would leave cells in distinguishable intracellular ^13^C-labelling states, and that ATP synthase inhibition would prove metabolically separable from upstream blockade by preserving a distinct pattern of oxidative and anaplerotic carbon redistribution. Three features of the design constrain what may be claimed from it. First, the exposures are matched on cellular response rather than on duration or target occupancy: four compounds were applied for 24 h and 3-NP for 12 h, the duration required to reach the same viability endpoint, so the five conditions are compound-defined perturbations at an equivalent degree of cellular stress rather than duration-matched Complex I–V inhibition states. Second, although each compound is defined by a nominal molecular target, none is fully selective for that target; Section 3.8 appraise the documented additional activities of each. Third, the tracer was supplied after the inhibitor had been removed, so the labelling data describe the metabolic state that persists after a complex-targeted insult rather than flux measured during ongoing inhibition. We therefore refer throughout to compound-defined post-exposure metabolic states, and Section 2.4 presents three readouts acquired within the labelling window itself that bear directly on whether the consequences of exposure persisted through tracing.

## 2. Results

### 2.1. Global Structure of the Intracellular ^13^C Metabolome

**Figure 1:**
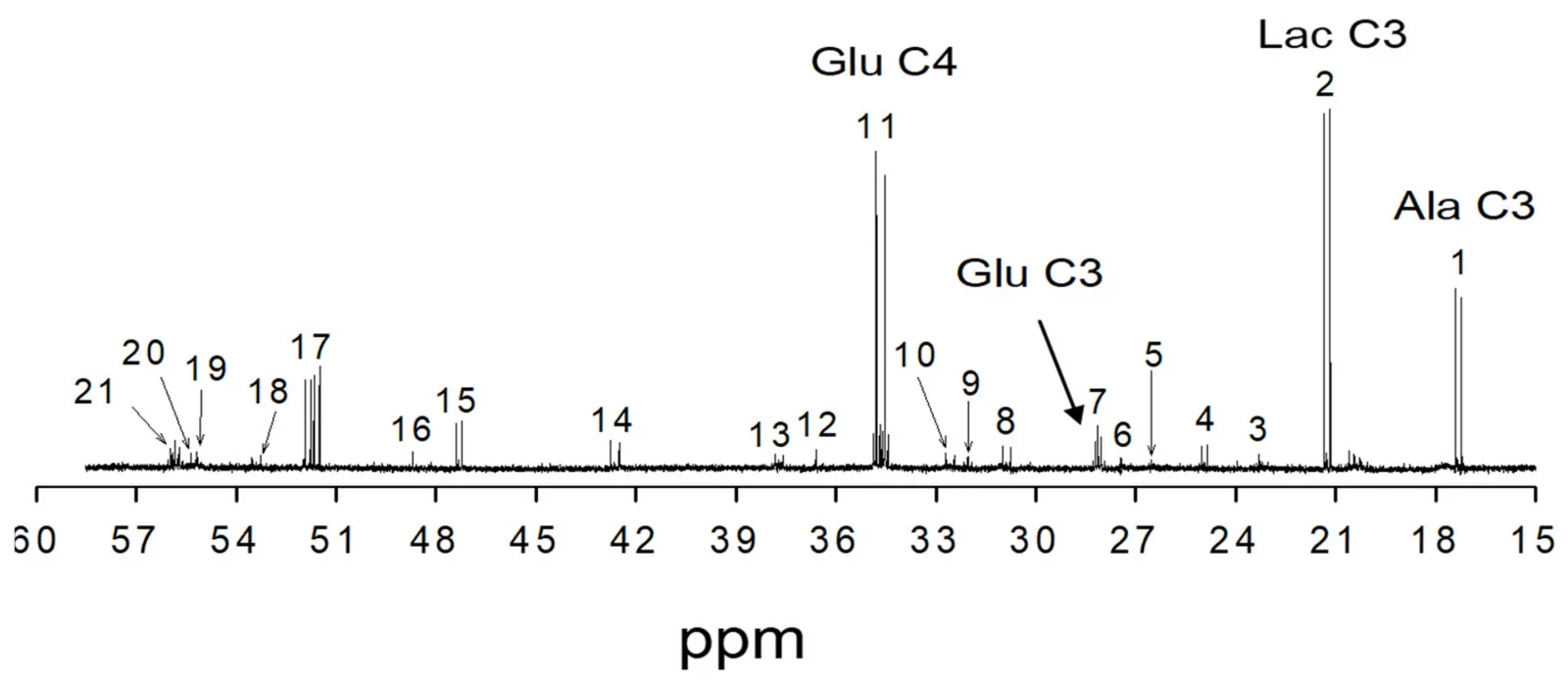
Representative ^13^C-NMR spectrum of SH-SY5Y neuroblastoma cell metabolites. Carbon positions follow IUPAC numbering throughout, so that GABA C1 is the carboxyl carbon and GABA C4 the amine-bearing carbon. Numerals: 1, alanine C3; 2, lactate C3; 3, GABA C3; 4, proline C4; 5, glutamine C3; 6, glutathione C3; 7, glutamate C3; 8, proline C3; 9, glutamine C4; 10, glutathione C4; 11, glutamate C4; 12, taurine C2; 13, aspartate C3; 14, glycine C2; 15, citrate C2/C4; 16, taurine C1; 17, alanine C2; 18, aspartate C2; 19, glutamine C2; 20, glutathione C2; and 21, glutamate C2.

Figure 1 presents a representative ^13^C-NMR spectrum of SH-SY5Y neuroblastoma cell metabolites acquired in the absence of ETC inhibition; the numbered assignments are listed in the legend.

**Figure 2:**
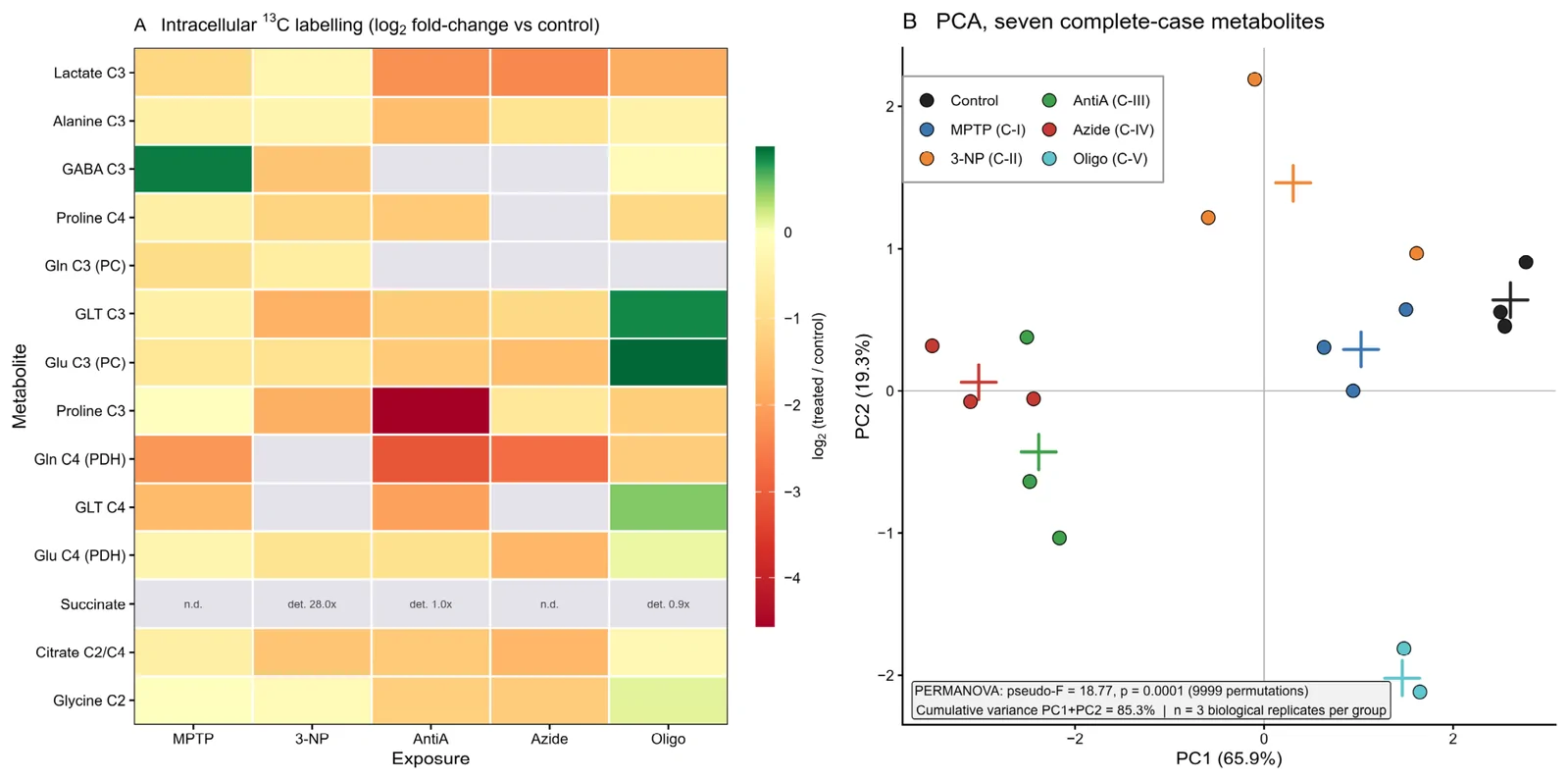
Global intracellular metabolite changes across complex-specific ETC inhibition. (**A**) Heatmap of intracellular ^13^C-labelled metabolites. Cells are coloured by log2 fold-change relative to control. Empty cells indicate metabolite not detected in that condition. (**B**) PCA scores plot of the same matrix; filled circles are individual biological replicates and crosses are group centroids. The ordination is descriptive and displays the structure formally tested by PERMANOVA; it is not evidence of pairwise separation between individual exposure groups. Variance explained by PC1 and PC2 is indicated on the axes (cumulative 85.3%). The ordination uses the seven complete-case metabolites defined in Section 4.8; group separation was tested by PERMANOVA (pseudo-F = 18.77; *p* = 0.0001).

Global intracellular metabolite changes across complex-specific inhibitions are shown in Figure 2A. Because the five exposures share a single vehicle-control triplicate, the multivariate structure of the dataset was tested directly rather than inferred from the ordination. Permutational multivariate analysis of variance on the log2 labelling matrix, which makes no distributional assumption and derives its null distribution by permuting the 18 samples across the six groups, demonstrated a significant overall effect of exposure group on the multivariate labelling matrix (pseudo-F = 18.77; *p* = 0.0001; 9999 permutations). Principal component analysis of the same matrix showed visually distinct group structure on the PC1-PC2 plane, with the control replicates and each exposure group occupying separate regions of the ordination (Figure 2B). The ordination is descriptive: it displays the structure that PERMANOVA tests, and no pairwise PERMANOVA was performed, so we make no claim that every pair of exposure groups differs significantly. PC1 and PC2 explained 65.9% and 19.3% of total variance, respectively (85.3% cumulative). The ordination was computed on the seven metabolites detected in all three replicates of all six groups (lactate C3, alanine C3, glutamate C3, glutamate C4, glycine C2, citrate C2/C4 and alanine C2); metabolites undetected in one or more groups were excluded rather than imputed, so that no substituted value contributes to the component structure (Section 4.8). The intracellular log2 fold-change heatmap (Figure 2A) revealed both shared features (broad reduction in glycolytic and oxidative-entry labels under most inhibitors) and inhibitor-specific deviations (succinate accumulation under 3-NP; selective elevation of glutamate C3 under oligomycin).

### 2.2. Complex-Specific Intracellular Labelling Phenotypes

Table 1 reports percent-of-control values and multiplicity-adjusted *p*-values for every detectable intracellular metabolite, including glycolytic end-product labelling (lactate C3, alanine C3) and glutamate positional labelling (C4, PDH-associated; C3, PC-associated) in each inhibitor condition. All comparisons are Dunnett many-to-one contrasts against the shared vehicle control on log2-transformed values, with the error variance pooled across all six groups (Section 4.8). Inference, in this study, is hierarchical. The prespecified mechanistic endpoints are the PDH-associated glutamate C4 readout, the PC-associated glutamate C3 readout, the relationship between them, and the PC and PDH enzyme-capacity assays; the conclusions rest on these. All remaining resonances, including lactate, alanine, citrate, glycine, glutamine, glutathione, proline, taurine and aspartate positions, are exploratory metabolite-wide endpoints reported for completeness, and borderline results among them are not treated as established effects. Table 2 sets out the precursor route that each resonance reports. Two features of these assignments bear on how the per-condition results below should be read, and are stated here rather than only in the note to Table 2. First, the route assignments are first-turn relationships: carbon entering through pyruvate dehydrogenase labels glutamate C4 and C5, whereas carbon entering through pyruvate carboxylase labels glutamate C2 and C3. Second, recirculation through the symmetric intermediates succinate and fumarate redistributes label between these positions, and asymmetry between the C2/C3 and C4/C5 pairs is expected to persist only through the first two to three turns of the cycle [19]. At the 24 h labelling endpoint used here, the system has passed well beyond that point, so scrambling must be assumed to be substantially complete. No scrambling correction was applied, and none is possible from a single uniformly labelled glucose tracer at isotopic steady state. The positional contrasts reported below are therefore integrated, pathway-associated indicators of the relative weight of oxidative versus anaplerotic entry, and are not estimates of flux through either route; where the text refers to PDH-associated or PC-associated labelling, that qualification is intended throughout.

MPTP exposure (Complex I-related metabolic phenotype): MPTP produced moderate decreases in lactate C3 (48.3 +/− 10.7% of control, *p* = 0.008), glutamate C3 (61.5 +/− 8.5%, *p* = 0.030), proline C4 (72.9 +/− 5.6%, *p* = 0.029), Gln C4 (22.4 +/− 14.8%, *p* = 0.003) and GLT C4 (33.5 +/− 5.4%, *p* < 0.001). Under the shared-control analysis, the MPTP reductions in alanine C3 (73.6 +/− 12.6%, *p* = 0.259), glutamate C4 (82.1 +/− 4.2%, *p* = 0.476) and citrate C2/C4 (72.1 +/− 5.0%, *p* = 0.497) are no longer individually significant; however, all three remain directionally consistent with the other conditions. In the same condition, GABA C3 rose to 186.5 +/− 3.5% of control, but neither the omnibus test for that metabolite (*p* = 0.064) nor the Dunnett contrast (*p* = 0.069) reaches significance, and the control triplicate for GABA C3 is the most dispersed in the dataset (coefficient of variation 48.5%). That increase is therefore reported as an observation and is not treated as an established effect; its interpretation in Section 3.2 is qualified accordingly. MPTP is the weakest of the five exposures under this analysis, as it was under the unadjusted analysis.

3-NP exposure (Complex II-related metabolic phenotype): 3-NP was distinguished by the appearance of ^13^C-labelled succinate, undetectable in control extracts, together with reduced proline C4 (45.4 +/− 2.1%, *p* < 0.001), glutamate C3 (56.8 +/− 15.2%, *p* = 0.010), glutamate C4 (57.4 +/− 18.5%, *p* = 0.004) and citrate C2/C4 (37.6 +/− 13.5%, *p* = 0.003). A labelling pattern of this shape, in which glucose-derived succinate accumulates while downstream TCA labels contract, is consistent with succinate accumulation and disruption of TCA metabolism associated with Complex II inhibitor exposure, and it matches the ^13^C-NMR observation of Bakken and colleagues in cultured astrocytes, where labelled succinate was undetectable under control conditions and increased dose-dependently with 3-NP [20].

Antimycin A exposure (Complex III-related metabolic phenotype): Antimycin A caused the broadest suppression of intracellular ^13^C labelling, decreasing every metabolite in Table 1 for which a comparison against control could be computed. Among the larger changes, lactate C3 fell to 20.9 +/− 2.9% (*p* < 0.001), alanine C3 to 34.5 +/- 5.5% (*p* < 0.001), glutamate C3 to 40.2 +/− 12.4% (*p* < 0.001), glutamate C4 to 55.3 +/− 10.4% (*p* = 0.003), citrate C2/C4 to 40.3 +/− 1.6% (*p* = 0.007), glycine C2 to 43.7 +/− 3.2% (*p* < 0.001) and, among the exploratory endpoints, alanine C2 to 34.3 +/− 7.2% (*p* < 0.001; Supplementary Table S1).

Sodium azide exposure (Complex IV-related metabolic phenotype): Azide exposure produced the strongest reduction in the PDH-associated glutamate C4 labelling signal, which fell to 31.9 +/− 3.7% of control (*p* < 0.001; Hedges’ g = −8.3). Further decreases occurred in lactate C3 (19.4 +/− 2.7%, *p* < 0.001), alanine C3 (58.7 +/− 3.8%, *p* = 0.028), glutamate C3 (34.5 +/− 4.2%, *p* < 0.001), citrate C2/C4 (32.2 +/− 13.8%, *p* < 0.001), glycine C2 (41.0 +/− 11.1%, *p* < 0.001) and, among the exploratory endpoints, alanine C2 (22.6 +/− 3.0%, *p* < 0.001; Supplementary Table S1).

Oligomycin exposure (Complex V-related metabolic phenotype): oligomycin diverged most sharply from the other four conditions. The PC-associated glutamate C3 readout rose to 197.7 +/− 8.1% of control (*p* = 0.004; Hedges’ g = 17.8, 95% CI 5.3 to 30.2), whereas the PDH-associated glutamate C4 readout held at 107.2 +/− 5.0% (*p* = 0.971). GLT C3, likewise, rose to 180.2 +/− 30.7%; however, under the shared-control adjustment, that contrast falls just short of significance (*p* = 0.062), and citrate C2/C4 remained comparatively preserved at 87.0 +/− 13.7% (*p* = 0.951). In the same condition, intracellular lactate C3 and proline C3 stayed below control, at 28.4 +/− 9.5% (*p* < 0.001) and 42.2 +/− 3.4% (*p* < 0.001), as did the exploratory alanine C2 endpoint (32.3 +/− 6.9%, *p* < 0.001; Supplementary Table S1); the reduction in alanine C3 to 75.6 +/− 7.0% is not significant under the adjusted analysis (*p* = 0.349). The divergence between preserved glutamate C4 and nearly doubled glutamate C3 is the largest effect in the dataset by a wide margin and is the one result that the present design supports with two methodologically independent measurements.

### 2.3. Extracellular Labelling and Pathway-Associated Ratios

**Figure 3:**
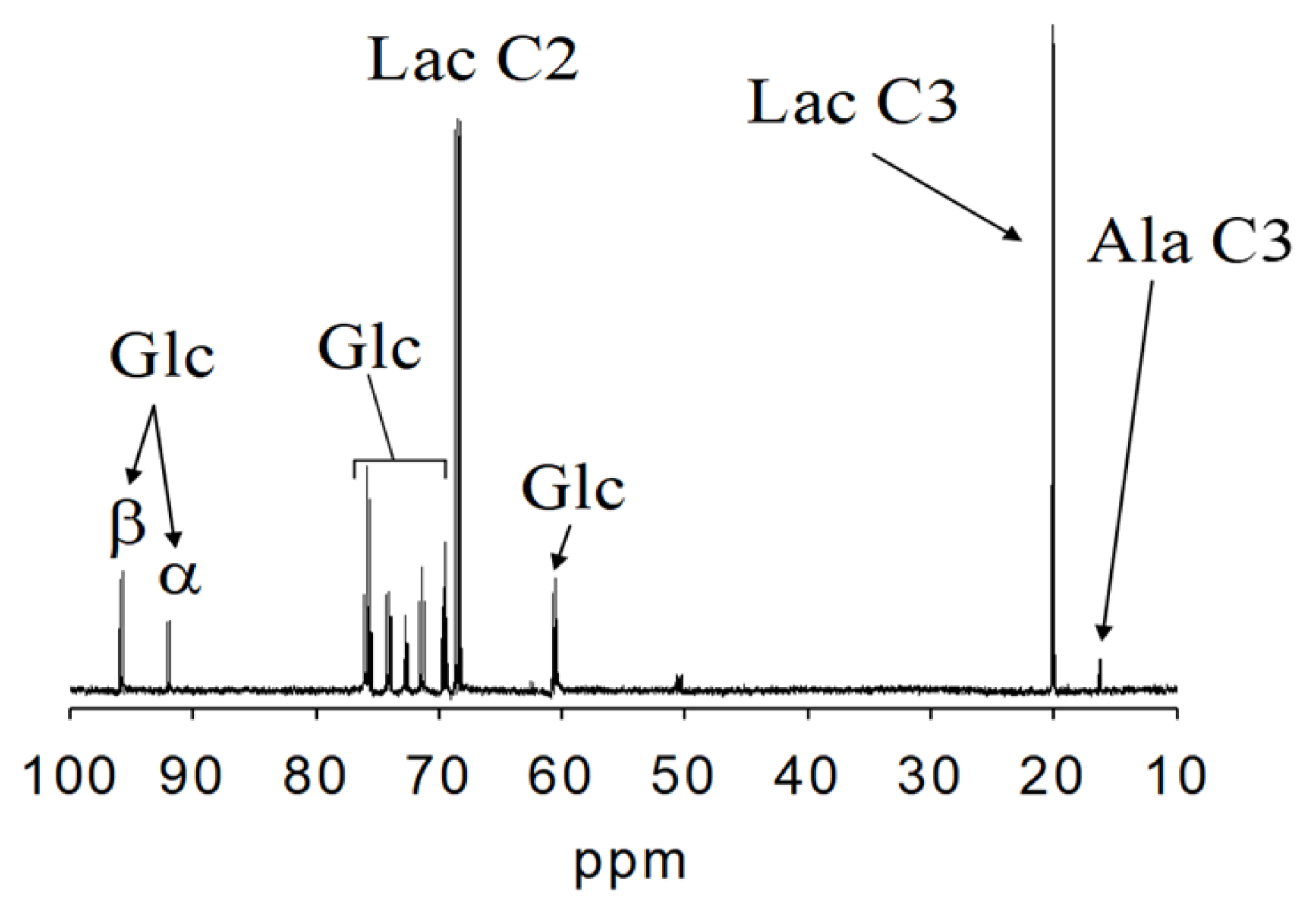
^13^C-NMR spectrum of the growth medium.

Extracellular labelling changes also differed by inhibitor. Relative to the control, the glucose α/β resonances changed by −52.4/−52.2% for MPTP, +111.6/+103.1% for 3-NP, −44.3/−46.1% for antimycin A, +75.3/+73.8% for sodium azide and −38.7/−36.5% for oligomycin. Lactate C3 in the medium rose with 3-NP (+60.6%), antimycin A (+73.8%) and sodium azide (+140.3%), but not with MPTP (−0.9%) or oligomycin (−6.0%). Alanine C3 in the medium rose most strongly with sodium azide (+95.5%) and oligomycin (+224.3%) (Table 3). A representative ^13^C-NMR spectrum of the conditioned growth medium, showing the glucose, lactate and alanine resonances quantified in Table 3, is presented in Figure 3.

**Figure 4:**
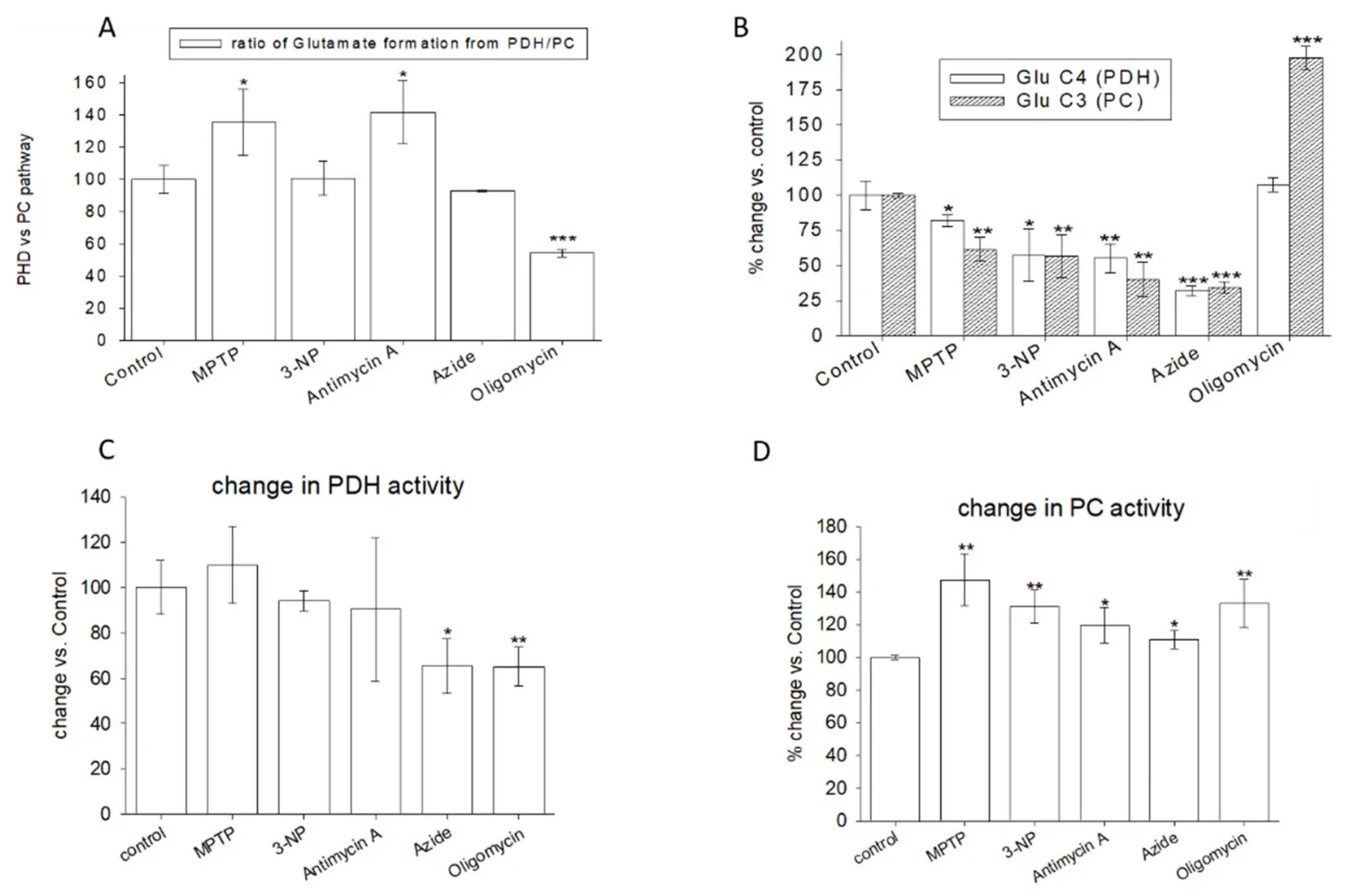
Glutamate C4/C3 positional labelling via PDH and PC pathway. (**A**) PDH/PC glutamate ratios vs. control. (**B**) Glutamate C4 (PDH-associated) and glutamate C3 (PC-associated) positional labelling. (**C**) Changes in PDH activities upon ETC inhibition. (**D**) Changes in PC activities upon ETC inhibition. Bars are mean ± SD (n = 3 biological replicates) expressed relative to the shared vehicle control. Each exposure was compared with the control by two-tailed, two-sample Student’s *t*-test assuming equal variance; *, *p* ≤ 0.05; **, *p* ≤ 0.01; ***, *p* ≤ 0.001. Unlike the positional-labelling data in Table 1, these comparisons are unadjusted for the five contrasts made against the shared control (Section 4.8); the enzyme-capacity panels are therefore read as directional corroboration of the labelling result rather than as independent significance claims.

The glutamate C4/C3 positional ratio distinguished oligomycin still further from the other conditions: Complexes I to III gave ratios of 0.93 or above, whereas oligomycin gave approximately 0.54 (Figure 4A,B).

The enzyme assays were directionally concordant with the positional labelling. Under oligomycin, PC activity rose to approximately 138% of control (*p* < 0.01), while PDH activity fell to approximately 60% (Figure 4C,D).

### 2.4. Readouts Acquired Within the Labelling Window

Because [U-^13^C_6_]glucose was supplied only after the inhibitor had been washed out, every labelled molecule reported here was synthesised during the 24 h tracing period. Three features of the dataset therefore report on the metabolic state of the cells during that window rather than during exposure.

First, ^13^C-labelled succinate was detected in all three 3-NP replicates and in no control extract, at approximately thirty-fold the normalised intensity seen under antimycin A or oligomycin and with no signal at all under MPTP or azide. Because succinate was absent from control, no ratio to control or significance test is available for this comparison (Table 1, footnote a), and the observation is qualitative. It is nonetheless informative: succinate carrying glucose-derived ^13^C can only have been generated during the labelling period, and its appearance implies that it was not being consumed at the control rate over that same period, consistent with succinate dehydrogenase remaining inactivated after washout as expected for a covalent suicide inactivator (Section 3.3). The same qualitative pattern, labelled succinate undetectable in control and rising with 3-NP exposure, has been reported by ^13^C NMR in cultured astrocytes [20]. The assignment of this resonance to succinate rather than to a GABA carbon, which differs from the annotation carried in the primary integral tables, is documented in Section 4.4.

Second, extracellular ^13^C-lactate rose during the labelling window under antimycin A (+73.8%) and sodium azide (+140.3%), and extracellular ^13^C-alanine rose under azide (+95.5%) and oligomycin (+224.3%) (Table 3). Increased export of glucose-derived three-carbon end-products into the medium during tracing is consistent with cytosolic NAD+ regeneration through lactate dehydrogenase when mitochondrial NADH oxidation is constrained, and it was measured contemporaneously with, not before, the labelling period. Because the normalisation and the multiplicity treatment of the medium data are not fully specified (Table 3), we use this observation only as supporting evidence that the cells had not returned to the control state during tracing, and not as independent mechanistic evidence for a redox interpretation.

Third, the pyruvate carboxylase and pyruvate dehydrogenase activities in Figure 4C,D were measured on extracts harvested at the end of the labelling period and remained divergent from control at that point, with PC at approximately 138% and PDH at approximately 60% of control under oligomycin. The enzymatic remodelling established during exposure was therefore still present at harvest.

These three observations are internal to the existing dataset and are not a substitute for condition-matched respirometry. They constrain the interpretation in a specific and limited way: they indicate that the cells had not returned to the control metabolic state by the end of the labelling window, without establishing which molecular lesion was still occupied. Their implications and limits are considered further in Section 3.9 and Section 3.10.

## 3. Discussion

### 3.1. Principal Observations and Interpretive Frame

The principal observation of this study is that perturbation at different mitochondrial respiratory nodes does not produce a single metabolic failure state, and the clearest instance of that heterogeneity is the Complex V condition, in which oligomycin exposure preserved PDH-associated glutamate C4 labelling while nearly doubling PC-associated glutamate C3, with concordant changes in directly measured pyruvate carboxylase and pyruvate dehydrogenase capacity (Section 3.6). That combination separates the Complex V condition from all four upstream conditions and is the one result supported by two independent classes of measurement (Section 3.6). Each lesion imposed a distinct pattern of glucose-derived ^13^C distribution with the clearest contrasts in glycolytic end-product labelling, in glutamate positional labelling and in the preservation or contraction of TCA-linked metabolites.

Throughout this discussion, the term “post-exposure metabolic state” denotes the steady-state ^13^C distribution measured during a 24 h tracing period that followed, rather than coincided with, inhibitor treatment. The patterns reported here are accordingly interpreted as post-exposure metabolic states and not as direct flux measurements obtained during ETC blockade [21,22,23,24,25]. We also distinguish two levels of inference. Observed labelling refers to the steady-state ^13^C fractional enrichment measured at individual carbon positions after 24 h of incubation with [U-^13^C_6_]glucose (Table 3). Pathway-associated interpretation refers to the assignment of those positional patterns to oxidative (PDH) or anaplerotic (PC) entry routes into the TCA cycle, following the established carbon-routing logic in which glutamate C4 enrichment reports acetyl-CoA-derived oxidative entry and glutamate C3 enrichment reports oxaloacetate-derived anaplerotic entry [13,14,16,17]. At a 24 h steady-state endpoint, however, these positional labels integrate over several processes that the present design cannot deconvolve: multi-turn TCA-cycle recycling; α-ketoglutarate-glutamate exchange across the inner mitochondrial membrane; reductive carboxylation of α-ketoglutarate by IDH1/IDH2 [26,27]; partial or reversed TCA fluxes; and dilution of positional enrichment by glutaminolytic carbon, which is unlabelled in this tracer design and varies across conditions with mitochondrial state. The C4-versus-C3 contrast is therefore an integrated pathway-associated indicator, and its directional concordance with the orthogonal PC and PDH enzyme-activity assays constitutes the strongest internal-consistency argument the design affords. Resolving reductive carboxylation, PC-specific anaplerosis and oxidative TCA contributions independently would require orthogonal labelling with [U-^13^C_5_]glutamine, position-specific glucose tracers such as [3,4-^13^C2]glucose, or ^13^C-bicarbonate at shorter time courses.

### 3.2. Complex I Inhibition (MPTP)

Under MPTP we observed concurrent reductions in lactate C3, alanine C3, glutamate C3 and glutamate C4, together with a selective increase in GABA C3. MPTP is a pro-toxin: the active Complex I inhibitor is its monoamine oxidase B-derived metabolite MPP+, which inhibits NADH:ubiquinone oxidoreductase at a site overlapping the rotenone/piericidin A binding site [28,29]. Because MAO-B activity in undifferentiated SH-SY5Y is limited, the effective intracellular Complex I lesion produced by 1 mM MPTP should be both partial and time-dependent [7,8]. Xicoy et al. [7] documented, in their systematic review, that undifferentiated SH-SY5Y expresses MAO-B at substantially lower levels than the differentiated form, so that conversion of MPTP to MPP+ in undifferentiated cells is incomplete; Risiglione et al. [8] confirmed, by high-resolution respirometry in differentiated SH-SY5Y, that direct MPP+ exposure produces a measurable Complex I-linked respiratory defect once the MAO-B step is bypassed, which brackets the upper limit of the lesion that 1 mM MPTP could produce in the undifferentiated state used here. The labelling pattern attributed to Complex I should therefore be read as a partial block of NADH oxidation and not as a clean Complex I knock-out comparable to piericidin or rotenone. Under the shared-control analysis adopted here (Section 4.8), several MPTP comparisons that were individually significant on unadjusted pairwise testing no longer are, including citrate C2/C4 (*p* = 0.497), alanine C2 (*p* = 0.083), alanine C3 (*p* = 0.259) and glutamate C4 (*p* = 0.476); the GABA C3 increase likewise does not survive (omnibus *p* = 0.064, Dunnett *p* = 0.069). What remains significant under MPTP is lactate C3 (*p* = 0.008), glutamate C3 (*p* = 0.030), proline C4 (*p* = 0.029), Gln C4 (*p* = 0.003) and GLT C4 (*p* < 0.001). The MPTP condition therefore rests on a narrower set of effects than the original analysis implied, and it is the weakest of the five conditions both statistically and pharmacologically (Section 3.8). We interpret it solely as a partial Complex I-associated constraint on NADH oxidation.

The pattern is most consistent with impaired NADH oxidation that constrains oxidative glucose use without abolishing carbon entry. Constraining Complex I activity in cultured cells lowers the cytosolic NAD+/NADH ratio, which in turn limits aspartate production and restricts biosynthetic competence [1,2,3,4]. Loss of NADH re-oxidation also propagates back to glycolytic intermediates as cytosolic NAD+ becomes limiting [1,2,3]. Consistent with this, Basu and Blair showed that Complex I inhibition by rotenone in SH-SY5Y reduces [U-^13^C_6_]glucose-derived ^13^C-acetyl-CoA from 23% to 5% without altering total acetyl-CoA [11]. The reduction in glutamate C4 observed here under MPTP agrees with that finding, since glutamate C4 reports PDH-derived acetyl-CoA entering the TCA cycle. The concurrent increase in GABA C3 identifies which carbon pool is redistributed. Glutamate decarboxylase removes the α-carboxyl (C1) of glutamate and leaves the remaining skeleton intact, so that under IUPAC numbering glutamate C3 becomes GABA C3 and glutamate C4 becomes GABA C2. The GABA C3 resonance consequently reports the same PC-associated anaplerotic carbon as glutamate C3, one step beyond decarboxylation. Under MPTP the two labels move in opposite directions: glutamate C3 fell to 61.5 ± 8.5% of control while GABA C3 rose to 186.5 ± 3.5%. Simple contraction of the glutamate pool cannot produce that divergence; redistribution of anaplerotically derived carbon out of glutamate and into GABA can.

The redox argument developed above supplies a mechanism for this redistribution without invoking any change in glutamate decarboxylase activity. GABA is disposed of through the GABA shunt: GABA aminotransferase converts it to succinic semialdehyde, and succinic semialdehyde dehydrogenase then oxidises that intermediate to succinate. This final step is NAD+-dependent, reducing NAD+ to NADH as it proceeds [30]. A Complex I lesion that lowers the NAD+/NADH ratio should therefore slow GABA disposal at precisely that step, allowing glucose-derived GABA to accumulate while the upstream glutamate pool contracts. The reading is also consistent with the wider dataset, since the same shunt delivers carbon to succinate and hence to Complex II, the step targeted directly in the 3-NP condition (Section 3.3). This reading must now be weakened rather than merely qualified. The GABA C3 increase does not survive the shared-control analysis (omnibus *p* = 0.064; Dunnett *p* = 0.069), and the control triplicate for this resonance has the largest dispersion of any row in Table 1 (coefficient of variation 48.5%), so the apparent divergence between glutamate C3 and GABA C3 under MPTP rests on a control estimate that is itself poorly determined. The present measurements also cannot separate increased GABA synthesis from decreased GABA disposal. We therefore retain the GABA shunt argument only as a hypothesis that is consistent with the redox reading of the MPTP condition and that would require direct measurement of GABA, succinic semialdehyde and the NAD+/NADH ratio to test; it is not offered as a finding of this study, and none of the conclusions depends on it.

### 3.3. Complex II Inhibition (3-NP)

**3**-NP is a suicide inactivator of succinate dehydrogenase (SDH), originally identified as the toxic principle of Indigofera endecaphylla [31]. The structural basis is the covalent modification of catalytic-base Arg-297 in the SDH-A subunit after enzymatic oxidation of 3-NP to 3-nitroacrylate, demonstrated crystallographically by Huang and colleagues [21]; the inactivation is therefore irreversible on the timescale of the present experiment. Because succinate dehydrogenase is simultaneously a TCA enzyme and the respiratory Complex II, 3-NP interrupts forward TCA carbon flow at the succinate-to-fumarate step and limits electron transfer to ubiquinone in the same lesion.

A labelling situation suggesting succinate accumulation and disruption of TCA metabolism associated with Complex II inhibitor exposure was observed: the appearance of ^13^C-labelled succinate was accompanied by reduced citrate C2/C4 (37.6%, *p* = 0.003), reduced glutamate C3 (56.8%, *p* = 0.010) and C4 (57.4%, *p* = 0.004), and reduced proline C4 (45.4%, *p* < 0.001), a combination consistent with a TCA cycle that is simultaneously interrupted and oxidatively constrained. Glycolytic compensation under this lesion most plausibly reflects the need to regenerate cytosolic NAD+ once mitochondrial oxidation becomes inefficient.

In genetic models of chronic SDH loss, cells become dependent on PC-mediated anaplerosis to sustain aspartate biosynthesis, and ^13^C labelling demonstrates increased flux of glucose-derived carbon into oxaloacetate and aspartate pools [32,33,34]. Cheng et al. showed further that PC induction can substitute for glutamine-dependent anaplerosis when the latter is suppressed [35]. The present dataset shows no such compensation: PC-associated glutamate C3 labelling fell under 3-NP rather than rose. We attribute this divergence to exposure duration, since the 3-NP treatment here lasted 12 h, whereas the transcriptional and enzymatic remodelling that establishes PC dependence in chronic models develops over cell generations [32,33,34].

### 3.4. Complex III Inhibition (Antimycin A)

Antimycin A binds the Qi site of Complex III and prevents reduction of ubiquinone by cytochrome b, leaving the upstream Q-pool reduced. Quantitative kinetic studies establish antimycin as a slow, tight-binding inhibitor of cytochrome bc1 with a K_i_ of 0.033 nM [22] and a low-picomolar dissociation constant at Centre N [23], so inhibitory occupancy persists into the labelling period after extracellular washout. The reduced Q-pool generates superoxide at the Qo site by stabilising a reactive semiquinone, and the rate of superoxide production varies sigmoidally with the reduction state of cytochrome b566 [36,37]. Sustained Complex III inhibition in intact human HEK293 cells elevates cytosolic H2O2 without necessarily producing overt oxidative damage on the timescales examined [38,39], and ETC-inhibitor-driven mitochondrial ROS suffices to trigger downstream cell-death programmes in cultured cells [40].

Antimycin A produced the broadest suppression of intracellular ^13^C labelling, decreasing every metabolite in Table 1 for which a comparison against control could be computed. This pattern is consistent with a severe combined limitation of carbon oxidation and electron transfer, in which the upstream Q-pool is heavily reduced and downstream cytochrome electron flow is blocked. Under such conditions, dehydrogenase activity throughout the matrix is disfavoured and fewer labelled intermediates remain available for the synthesis of glycine, alanine, citrate, glutamate and proline. The pattern is best described as a metabolic labelling pattern consistent with redox suppression rather than as a demonstration of altered redox state, since neither the NAD+/NADH ratio nor reactive oxygen species were measured directly here. We do not infer that ROS caused the broad suppression, an inference that would require direct ROS measurement. We note only that the conditions known to favour ROS generation at this complex [36,37,38,39,40] coincide here with the conditions of carbon-labelling suppression. Antimycin A has been reported to elevate mitochondrial reactive-oxygen-species production in SH-SY5Y cells specifically [41,42], thus indicating that the ROS-favouring conditions invoked here operate in the present neuronal-like background and not only in the non-neuronal systems cited above.

### 3.5. Complex IV Inhibition (Sodium Azide)

Under azide, the strongest reduction fell on PDH-associated glutamate C4 labelling (31.9% of control), accompanied by reduced citrate C2/C4, glycine C2 and alanine C2. The pattern fits a terminal-electron-acceptor block in which upstream redox carriers remain reduced and oxidative carbon handling is particularly disfavoured. Azide also differs from antimycin A in the selectivity of its labelling signature: azide produced the deepest loss of PDH-associated labelling, whereas antimycin A produced a broader and less selective contraction across multiple metabolite classes. We interpret this as loss of cytochrome c oxidase activity constraining acetyl-CoA-mediated entry disproportionately relative to other steps. Sodium azide at the concentration used here (2 mM) has been shown directly to lower mitochondrial membrane potential and viability in SH-SY5Y cells [43], which confirms that the azide condition reflects genuine functional Complex IV impairment in this background. Furthermore, although azide is conventionally described as freely reversible, chronic exposure produces an irreversible loss of cytochrome c oxidase activity through holoenzyme dissociation [44]. The Complex IV phenotype therefore reflects sustained loss of terminal-oxidase function carried into the labelling window rather than full recovery of activity upon washout.

### 3.6. Complex V Inhibition (Oligomycin)

Oligomycin binds the c-ring of mitochondrial F1Fo-ATP synthase and blocks proton translocation through the Fo sector [45,46]. The consequence in intact cells is an ATP-restricted state in which proton-motive force builds up but cannot be discharged through ATP synthesis. Sub-micromolar potency at the c-ring (IC_50_ ≈ 100 nM for yeast F_1_F_o_ ATP hydrolysis; ≤1.25 µM for near-complete inhibition of ATP synthesis in human mitoplasts [24]) combined with extensive partitioning into the inner mitochondrial membrane is consistent with persistent inhibition at the 1 µg/mL (~1.27 µM) working concentration used here.

The Complex V phenotype was the most distinct of the five. Glutamate C4 was preserved near control (107.2%; *p* = 0.971) while glutamate C3 nearly doubled (197.7%; *p* = 0.004; Hedges’ g = 17.8) and GLT C3 rose to 180% of control (*p* = 0.062). The glutamate C3 effect is the largest in the dataset and is substantially above the estimated sensitivity threshold for this design (Section 4.8). That magnitude reflects both a large difference in group means and unusually small within-group variability across three independent biological replicates; with n = 3 per group it is not by itself evidence of broad reproducibility, and the weight we place on this result rests additionally on the concordant direction of the independent PC and PDH capacity measurements. Directly measured PC enzyme activity increased to approximately 138% of control under oligomycin, while PDH activity fell.

This is the result the study rests on, because it is the only one supported by two methodologically independent classes of measurement: an in-cell positional-labelling readout (glutamate C3 and C4) and an ex-cell enzyme-capacity readout (PC and PDH) obtained in parallel extracts from the same biological replicates. We read the combination as relative preservation of oxidative carbon entry together with a stronger PC-associated positional-labelling signature, concordant with increased PC assay capacity. We do not read it as a demonstration of increased PC flux. Three qualifications bound that interpretation. The enzyme assays report capacity under saturating cofactors (Section 4.6), so a rise in PC capacity does not establish that more carbon travelled the PC route in the intact cell; the glutamate C3 increase is the in-cell observation and the enzyme assay corroborates its direction. Pyruvate carboxylase is MgATP-dependent [47], so a condition that restricts oxidative ATP synthesis might be expected to constrain rather than expand PC-associated labelling; that tension resolves if cellular ATP is substantially restored during the labelling window, as occurs after ATP synthase blockade in other lineages where sustained glycolytic compensation returns ATP to pre-treatment levels within hours [48], and the inhibitor had in any case been removed before labelling began. We therefore read the Complex V condition as an ATP-restricted state that has partially rebalanced rather than an acutely ATP-depleted one. Finally, reductive carboxylation of alpha-ketoglutarate by IDH1/IDH2 is a documented route of citrate and acetyl-CoA generation in cells with impaired mitochondrial function [26,27,49] and in SDH-deficient cells [34], and [U-^13^C_6_]glucose alone cannot resolve reverse IDH flux at a 24 h endpoint, because glutamate C3 labelling from PC-derived oxaloacetate is not distinguishable from labelling generated by partial reverse-cycle activity on glucose-derived carbon. Direct demonstration would require ^13^C5- or 15N-labelled glutamine tracers and shorter time courses [26,27,49]. Our data therefore cannot establish, exclude or quantify a reductive component, and we claim none; the increased PC capacity is the only direct evidence bearing on the question, and on the present data oligomycin exposure generates an ATP-restricted state in which carbon redistribution favours a PC-associated positional-labelling signature.

### 3.7. Comparative Synthesis

Viewed across the five lesions, the data are most consistent with glycolytic compensation being not a single response but a family of responses, each shaped by the specific redox and topological consequences of the perturbation. When Complex I is impaired, cytosolic NAD+ regeneration through lactate or alanine is itself constrained, which accords with the established dependence of cytosolic NAD+/NADH balance on functional Complex I [1,2,3,4]. When the TCA cycle is interrupted physically at SDH, glycolytic intermediates accumulate, yet PC-driven compensation does not engage acutely, in contrast to chronic SDH-loss models [32,33,34,35]. When ATP synthesis is blocked downstream of electron transfer at Complex V, PC-associated labelling is favoured even without global preservation of glycolytic intermediates.

Comparative ^13^C-glucose flux analysis of mitochondrial and Parkinson’s disease toxins has been reported previously: Lei and colleagues showed that paraquat, rotenone, MPP+ and 6-OHDA induce divergent metabolic signatures in dopaminergic cells [12], and Zhang et al. reported that piericidin (Complex I), antimycin (Complex III) and oligomycin (Complex V) generate distinguishable signatures in primary RPE cells by LC-MS-based targeted metabolomics [18]. Consistent with the view that ETC inhibition reshapes rather than uniformly suppresses metabolism, Suciu and colleagues showed in human LUHMES neurons that the Complex I inhibitors rotenone, MPP+ and berberine, applied under conditions that did not deplete ATP, produced a coordinated metabolic signature dominated by oxidative stress, a marked rise in NADH and re-routing of carbon and nitrogen; they concluded that the toxicity of mitochondrial inhibitors reflects an ensemble of metabolic changes rather than a simple ATP-depletion defect [50]. That work used a different human neuronal model and addressed Complex I alone, but its conclusion parallels the complex-specific reshaping reported here. The present data extend these frameworks to all five ETC complexes within one matched neuronal-like cellular context and add positional ^13^C resolution, including the glutamate C3 versus C4 contrast that discriminates PC-route from PDH-route TCA entry [13,14,16,17]. Reduced citrate, glycine and glutamate labelling under Complexes II to IV is consistent with contraction of precursor supply for amino-acid and one-carbon-linked metabolism, whereas the oligomycin condition indicates relative preservation of carbon delivery into biosynthetically useful pools under energetic stress. These observations are compatible with the broader view that respiration supports cellular metabolism not only through ATP generation but also through maintenance of electron-acceptor availability and biosynthetic competence [1,2,3,4,5]. We conclude that ETC inhibition reshapes biosynthetic flux rather than simply suppressing it [6].

Methodologically, the present ^13^C NMR dataset and the LC-MS work cited above are complementary. ^13^C NMR resolves individual carbons within a single metabolite (glutamate C3 versus C4; GABA C2 versus C3; and alanine C2 versus C3) in one experiment and without prior chromatographic separation, at the cost of lower sensitivity and a smaller detectable metabolite set. MS-based isotope tracing offers in return higher sensitivity, mass-isotopomer distribution analysis, and resolution of the M+1, M+2 and M+3 isotopologues that NMR cannot access directly. That trade-off is narrowing: LC-MS/MS methods now resolve all thirty-two glutamate and sixteen aspartate isotopomers from roughly one per cent of the sample required for NMR, and can discriminate between routes that yield the same number of ^13^C labels [51]. The positional information used here is therefore not unique to NMR, and the practical case for the NMR readout rests on its ability to resolve multiple carbon positions in one unseparated measurement rather than on exclusivity. Combined NMR and MS analysis of parallel extracts is the natural orthogonal extension of the dataset reported here.

The present design does not include orthogonal perturbations at the same node, either chemically distinct inhibitors or genetic loss of a complex subunit, and this is a real limitation on the attribution of each phenotype to the complex named in its heading. Two independent lines of published evidence nevertheless bear on it. First, at Complex I, Giordano and colleagues compared rotenone, MPP+ and 6-hydroxydopamine in differentiated SH-SY5Y cells and found that the two Complex I-directed agents both produced immediate inhibition of basal and ATP-linked oxygen consumption with stimulation of glycolysis, while 6-hydroxydopamine, which is not a respiratory-chain inhibitor, did not; they also found that rotenone and MPP+ differed from one another in their effect on reserve capacity, so that two agents acting at the same complex do not produce identical cellular phenotypes [52]. That result supports the attribution of a Complex I-directed labelling pattern to the node rather than to the molecule, and at the same time cautions against expecting any single compound to stand for its complex. Second, at Complex II, the appearance of ^13^C-labelled succinate under 3-NP reported here reproduces, in a different neural cell type and with the same analytical method, the observation of Bakken et al. that labelled succinate is undetectable in control astrocytes and increases dose-dependently with 3-NP [20], and it is directionally concordant with the chronic genetic SDH-loss models already discussed [32,33,34], which diverge from the present data only in the direction expected from the difference in exposure duration (Section 3.9). These are literature concordances, not controls run in this system; the experiments that would convert compound-defined phenotypes into complex-attributed ones are listed in Section 3.11.

### 3.8. Inhibitor Polypharmacology and Its Consequences for Interpretation

None of the five compounds used here is fully selective for the respiratory complex it is conventionally used to inhibit, and several were applied at concentrations well above their on-target potency in order to reach the viability-matched endpoint described in Section 4.2. Any interpretation that attributes the observed labelling changes to respiratory inhibition alone is therefore incomplete. Table 4 sets out, for each compound, its nominal target and reported potency, the concentration used, the activities reported beyond that target and whether each of those activities is target-independent or arises downstream of the nominal lesion. That distinction matters, because activities of the second kind are part of the cellular response being described rather than alternatives to it.

Two entries in Table 4 are genuinely target-independent and are treated as alternative contributors rather than as downstream consequences. The first is the BH3-mimetic activity of antimycin A. Antimycin A binds the hydrophobic BH3-binding groove of Bcl-2 and Bcl-xL in competition with a Bak BH3 peptide, and the 2-methoxy derivative of antimycin A3 retains toxicity toward Bcl-xL-expressing cells and mitochondria while losing the ability to inhibit respiration [53]. This dissociation establishes that the activity does not require Complex III inhibition, and at the 7.3 µM working concentration used here, it cannot be excluded. The second is inhibition of store-operated calcium entry by oligomycin, which has an IC_50_ of approximately 2 µM, roughly 50-fold above the concentration that blocks mitochondrial ATP production, and which persists under voltage clamp with the cytosolic ATP concentration fixed by dialysis [57]. The 1.27 µM used here lies below that IC_50_ but not far below it. Because calcium is an activator of pyruvate dehydrogenase phosphatase [60], partial inhibition of store-operated calcium entry is a plausible alternative contributor to the reduced PDH activity measured under oligomycin, and we no longer attribute that reduction solely to the energetic consequences of ATP synthase blockade.

Other activities raised in connection with these compounds are, on the published evidence, downstream of the nominal lesion rather than parallel to it. Antimycin A accelerates c-Myc degradation, but it does so by generating reactive oxygen species from damaged mitochondria, which activate GSK3 and drive Thr-58 phosphorylation and proteasomal degradation of c-Myc [54]; the requirement for mitochondrial ROS places this effect downstream of Complex III inhibition. Similarly, opening of Cx43 hemichannels under metabolic inhibition accompanies ATP depletion and dephosphorylation of Cx43 [55], and the IRE1-dependent endoplasmic-reticulum stress signalling reported after oligomycin exposure [59], together with the Akt/mTORC2 and autophagic changes, are components of the integrated cellular response to energetic stress. If the objective is to describe the metabolic state that a Complex III or Complex V insult leaves behind, these processes are properly part of that state. They would confound the interpretation only under the stronger claim, which we no longer make, that the labelling changes report electron-transport inhibition in isolation.

A structural feature of the dataset limits how far compound-specific off-target pharmacology can account for the results. The five compounds are chemically unrelated and their reported additional activities do not overlap. A BH3 groove ligand, a covalent SDH inactivator, a haem-protein ligand, a calcium-channel blocker and a pro-toxin requiring enzymatic conversion. Yet antimycin A and azide converge on a shared contraction of oxidative-entry labels, and the conditions separate along interpretable axes in PCA rather than scattering. Convergent phenotypes arising from compounds whose off-target profiles are disjoint are more parsimoniously attributed to the property those compounds share, which is interruption of mitochondrial electron transfer or its coupling to ATP synthesis, than to five unrelated mechanisms producing coincidentally similar carbon redistribution. This is an argument from parsimony and not a control experiment. It does not exclude an off-target contribution to any individual phenotype, and it is weakest exactly where the reviewer of an earlier version of this work indicated it would be, namely for antimycin A, where a target-independent apoptotic mechanism is documented at a concentration comparable to the one used here.

### 3.9. Comparison of Experimental Designs Across Isotope-Tracing Studies of Respiratory Inhibition

The design used here differs in one important respect from most isotope-tracing studies with which these data invite comparison. In the majority of those studies the inhibitor is present throughout the labelling period, so the isotopologue pattern reports carbon handling during ongoing inhibition. Here the inhibitor was removed before the tracer was supplied, so the labelling pattern reports the state the cells occupy after the insult. Table 5 sets this out explicitly for the studies cited in Section 3.2, Section 3.3, Section 3.4, Section 3.5, Section 3.6 and Section 3.7. Reanalysis cannot remove the difference, and it means that quantitative labelling values obtained here are not directly comparable with values obtained under concurrent inhibition.

The design does, however, answer a different and legitimate question. Cells do not experience chronic mitochondrial impairment in neurodegeneration as an acute, continuously enforced block; the metabolic state that persists after an insult is itself of interest, and it is that state the present measurements describe. Reading the data this way also accounts for the one clear divergence from the published literature. In chronic genetic models of SDH loss, cells become dependent on pyruvate carboxylase for anabolism, and glucose carbon is redirected into oxaloacetate and aspartate [32,33,34]. Under acute 3-NP, we observed the opposite: PC-associated glutamate C3 labelling falling rather than rising. This is not a contradiction; instead, it is the expected difference between a lesion lasting hours and one lasting cell generations, since the transcriptional and enzymatic remodelling that establishes PC dependence takes time.

### 3.10. Neuropharmacological Significance

The practical implication of these data for neuropharmacology is that mitochondrial liability is not a single property. A compound that impairs Complex IV and a compound that impairs Complex V both reduce oxidative ATP production, yet the cells they leave behind differ in which carbon routes remain available: azide exposure leaves PDH-associated entry deeply suppressed, whereas oligomycin exposure leaves it near control while expanding PC-associated anaplerotic labelling. Two compounds classified together as mitochondrial toxicants would therefore be predicted to confer different residual biosynthetic capacity, and different sensitivity to a second metabolic challenge. This distinction is invisible to viability assays and to bulk metabolite abundance, both of which report the magnitude of an insult rather than its metabolic shape.

The oligomycin phenotype is the clearest illustration, and it is the observation for which the present design provides orthogonal support. Preserved PDH-associated glutamate C4 labelling with a nearly doubled PC-associated glutamate C3 readout, concordant with a measured rise in PC capacity and a fall in PDH capacity, indicates redistribution of pyruvate-derived carbon toward anaplerotic entry rather than a global contraction of carbon supply. Sustained cellular adaptation to ATP synthase blockade is well documented in other lineages, where oligomycin exposure elicits compensatory glycolytic gains and restoration of cellular ATP without cell death [48]; the present data indicate that in a neuronal-like background this adaptation also has an anaplerotic component detectable at the level of carbon routing. We frame this as a mechanistic hypothesis about metabolic resilience rather than a therapeutic claim, since neither flux nor any therapeutic endpoint was measured here.

Three boundaries should be stated explicitly so that this significance is not overread. Undifferentiated SH-SY5Y cells are a neuronal-like cell model and not a model of any neurological disease; MPTP is used here as a Complex I-directed pro-toxin and not as a Parkinson’s disease model, particularly given the limited MAO-B activity of the undifferentiated state (Section 3.2). None of the five compounds is a therapeutic agent, and nothing in these data speaks to therapeutic benefit or risk in patients. What the data support is narrower and still useful: a framework for anticipating that pharmacological insults at different mitochondrial nodes will generate non-equivalent metabolic states in neuronal cells, and a positional ^13^C-NMR readout capable of distinguishing them.

### 3.11. Limitations

Five limitations bound what these data can support. First, direct bioenergetic endpoints, oxygen consumption, ATP content, mitochondrial membrane potential, NAD+/NADH ratio, reactive oxygen species and individual respiratory-complex activities were not measured, so respiratory inhibition is inferred from the pharmacology of the compounds and from published measurements at comparable concentrations in this cell line rather than demonstrated in these cells; condition-matched respirometry is the principal follow-up experiment. Second, each complex was perturbed with one compound at one concentration and one time point, without dose-response, time-course, chemically distinct comparators or genetic models, and the Complex I lesion produced by MPTP in undifferentiated SH-SY5Y is partial because MAO-B activity in these cells is limited [7]; the published concordances that partially offset this are set out in Section 3.7 and are not a substitute for orthogonal controls run here. Third, the design is a single-factor, six-level layout with three biological replicates per group and one shared vehicle control, so power to detect small effects is low. A sensitivity analysis rather than a post hoc power calculation is the appropriate retrospective statement for a completed study [61,62], and is given in Section 4.8: at n = 3 per group, alpha = 0.05 and 80% power this design detects standardised effects of approximately d = 3.1 or larger, so the conclusions rest on large-magnitude changes, on effect sizes reported with confidence intervals, and on directional concordance with the orthogonal pyruvate carboxylase and pyruvate dehydrogenase activity assays (Figure 4C,D) rather than on the significance of individual metabolites. Fourth, the cells were undifferentiated SH-SY5Y, which carry a documented glycolytic bias relative to the retinoic acid-differentiated state, where mitochondrial membrane potential, reserve capacity and Complex IV subunit expression are all higher [9,10,63]. The oxidative and anaplerotic readouts on which this study depends therefore operate over a compressed dynamic range in the model used, and the differentiated state should be expected to widen rather than to reverse the contrasts reported here; the direction of this bias works against the findings rather than toward them, but it does mean that the absolute magnitudes are not transferable to mature neurons, the more so because respiratory and ATP-synthesis rates differ substantially even between mitochondria isolated from different regions of the same brain [64], and repetition in a differentiated or iPSC-derived neuronal model is a necessary extension. Fifth, the study reports carbon routing and not energy status. The design carries no measurement of adenylate pools, so it cannot say whether the post-exposure states described here are accompanied by a persistent change in energy charge, a question that bears directly on the interpretation of the Complex V phenotype in Section 3.6. ^1^H NMR can quantify ATP, ADP, AMP and the amino-acid pools in cell extracts simultaneously, and acquiring 1H spectra alongside the ^13^C spectra on the same extracts would be the natural way to add that layer. The perchloric-acid extraction used here was optimised for the stability of the carbon-skeleton metabolites rather than for adenylate preservation, and the extracts were lyophilised and stored before analysis, so we do not report adenylate data from them; a purpose-designed ^1^H study on freshly prepared extracts is identified as a direction for future work. The consequences of inhibitor selectivity and of the post-exposure labelling design are set out in Section 3.8 and Section 3.9 and are not repeated here.

## 4. Materials and Methods

**Figure 5:**
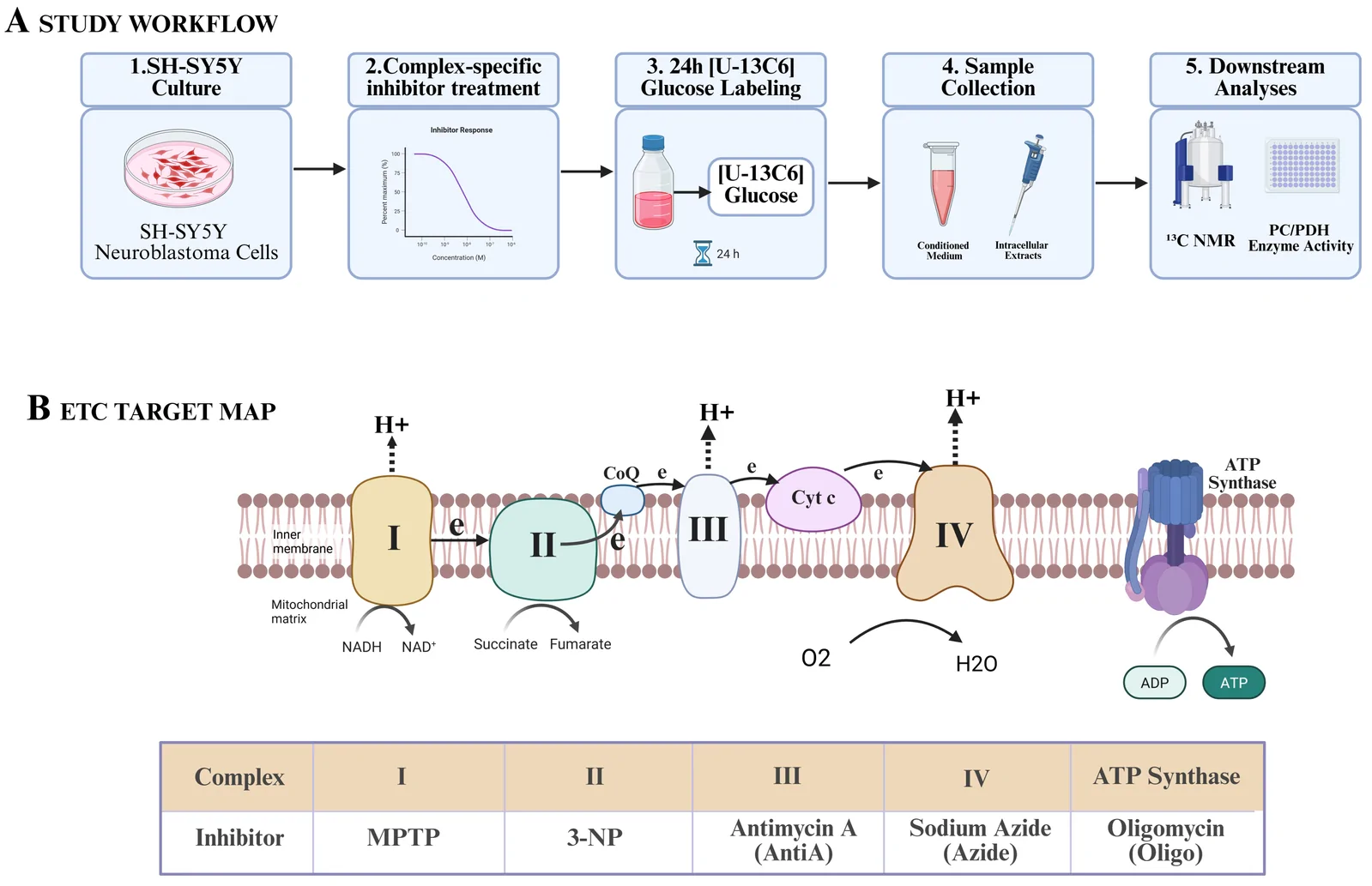
Experimental design and labelling logic. (**A**) SH-SY5Y cultures received complex-specific inhibitor treatment titrated to approximately 80% viability, followed by 24 h of [U-^13^C_6_]glucose labelling. Conditioned medium and intracellular extracts were then collected for parallel ^13^C NMR and PC/PDH enzyme-activity analyses. (**B**) ETC target map showing the inhibitor used at each respiratory complex: MPTP (Complex I), 3-NP (Complex II), antimycin A (AntiA, Complex III), sodium azide (Azide, Complex IV) and oligomycin (Oligo, Complex V). *Created in BioRender. Baykal, A. (2026). BioRender.com/xnnw3ns* and is reproduced under a BioRender publication licence.

An overview of the experimental design, complex-specific inhibitor targets and primary readouts is shown in Figure 5.

### 4.1. Materials

SH-SY5Y neuroblastoma cells were obtained from the American Type Culture Collection (Manassas, VA, USA). Dulbecco’s modified Eagle’s medium (DMEM), trypsin–MEM (0.05%) and penicillin/streptomycin were obtained from Mediatech Inc. (Manassas, VA, USA). Glucose-free DMEM and phosphate-buffered saline (PBS) were obtained from Gibco (Grand Island, NY, USA). Foetal bovine serum (FBS) was obtained from TC Biologicals (Tulare, CA, USA). Deuterium oxide (D_2_O, 99.996%), 3-(trimethylsilyl)propionic-2,2,3,3-d4 acid (TSP), deuterium chloride (DCl, 99% D), sodium deuteroxide (NaOD, 99.5% D), sodium formate-^13^C (99 atom % ^13^C), D-glucose-^13^C6 (99 atom % ^13^C), 1-methyl-4-phenyl-1,2,3,6-tetrahydropyridine (MPTP), 3-nitropropionic acid (3-NP), oligomycin, antimycin A and sodium azide were obtained from Sigma-Aldrich (St. Louis, MO, USA). Perchloric acid (70–72%) was obtained from JT Baker (Phillipsburg, NJ, USA).

### 4.2. Cell Culture and ETC Inhibition

SH-SY5Y cells were grown in DMEM containing 10% FBS, penicillin (100 units/mL), streptomycin (100 µg/mL) and non-essential amino acids (1% *v*/*v*) at 37 °C in a humidified atmosphere of 5% CO_2_/95% air. For each sample analysis, 108 cells were used. When cells reached approximately 90% confluence, samples were incubated with fresh culture medium containing 10% FBS, antibiotics and non-essential amino acids plus one of the following respiratory chain inhibitors: (i) 1 mM MPTP, a pro-toxin whose monoamine oxidase B-derived metabolite MPP+ inhibits NADH: ubiquinone oxidoreductase (Complex I) at a site that overlaps with the rotenone/piericidin A binding site [28,29]; (ii) 5 mM 3-nitropropionic acid (3-NP), an irreversible suicide inactivator of succinate dehydrogenase (Complex II) [21,31]; (iii) 4 µg/mL antimycin A, which binds the Qi site of Complex III and blocks electron transfer from cytochrome b to ubiquinone [36,37]; (iv) 2 mM sodium azide, an inhibitor of cytochrome c oxidase (Complex IV) that binds between the haem a3 iron and CuB at the oxygen-reduction site [56]; or (v) 1 µg/mL oligomycin, which binds the c-ring of mitochondrial F1Fo-ATP synthase (Complex V) and blocks proton-coupled ATP synthesis [45,46]. Incubation time for each complex-specific inhibition was 24 h, except for 3-NP (12 h), in order to maintain cell viability at approximately 80% (assessed by trypan blue exclusion). This biological-response-based normalisation accounted for differential inhibitor uptake, intracellular metabolism and inhibition kinetics across the five compounds. Concentrations were therefore anchored to a matched cellular response rather than to a fixed multiple of each compound’s in vitro potency, and for several compounds the resulting working concentration substantially exceeds that potency. This is most marked for antimycin A, where the 4 µg/mL (approximately 7.3 µM) used exceeds the reported Qi-site K_i_ of 0.033 nM [22] by roughly five orders of magnitude, and it is the principal reason that target-independent activities must be considered explicitly rather than assumed to be negligible (Section 3.8, Table 4). The requirement for high extracellular concentrations reflects a combination of limited cellular uptake over the exposure period, serum-protein binding of the lipophilic compounds, and, for MPTP, dependence on enzymatic conversion to the active species. Matching on viability rather than on nominal target occupancy was chosen so that the five conditions could be compared at an equivalent degree of cellular stress; the trade-off is that the compounds are not matched for target selectivity, which is addressed in Section 3.8. MPTP, 3-NP and sodium azide were prepared as aqueous stock solutions in sterile saline; antimycin A and oligomycin were prepared as concentrated stock solutions in absolute ethanol, with the final ethanol concentration in the working medium not exceeding 0.1% (*v*/*v*). A single vehicle-control triplicate was prepared, labelled and harvested alongside the exposure groups, and all five exposure conditions are compared against that one control triplicate; separate vehicle-matched controls for the saline- and ethanol-dissolved compounds were not run. The statistical analysis is constructed around this shared-control structure (Section 4.8), and the absence of a separate ethanol-vehicle arm means that a contribution of ≤0.1% ethanol to the antimycin A and oligomycin phenotypes cannot be excluded by an internal control, although effects of ethanol at this concentration on the labelling readouts reported here would be expected to be small relative to the effect sizes observed. Inhibitors were present during the pre-conditioning incubation (24 h, or 12 h for 3-NP) but were not re-added to the serum-free, glucose-free DMEM containing 5 mM [U-^13^C_6_]glucose used for the subsequent 24 h labelling period (see Section 4.3); the labelling phase therefore reads the metabolic state established during complex-specific inhibition. Because 3-NP is a covalent suicide inactivator of SDH that generates a 3-nitroacrylate-derived adduct on catalytic Arg-297 of the SDH-A subunit [21,31], antimycin A is a slow tight-binding Qi-site inhibitor of Complex III with picomolar affinity and slow dissociation kinetics [22,23,36], and oligomycin inhibits ATP synthase with sub-micromolar potency [24] and partitions extensively into the inner mitochondrial membrane at the c-ring [45], the inhibitory effect on Complexes II, III, and V is expected to persist into the labelling period. MPP+ is concentrated and retained in the mitochondrial matrix by the inner-membrane potential [25]. Sodium azide, by contrast, is conventionally regarded as reversible upon washout; chronic exposure, however, has been shown to cause an irreversible loss of cytochrome c oxidase activity through holoenzyme dissociation [44], and the Complex IV phenotype must be interpreted as the labelling pattern that emerges in cells that have just experienced 24 h of CcO inhibition rather than during ongoing inhibition.

Two points about this dosing scheme should be stated explicitly, because the exposures are not matched on target occupancy. Viability was monitored at every working concentration and was the quantity on which the doses were titrated: each inhibitor concentration was selected so that trypan-blue exclusion gave approximately 80% viability at the end of the exposure period, which is why the 3-NP exposure is 12 h, while the others are 24 h. Mitochondrial membrane potential was not measured in this study. The working concentrations nevertheless lie within the range used in published work on this cell line: 1 µM oligomycin and antimycin A at sub-micromolar-to-low-micromolar concentrations are the standard reagents of the mitochondrial stress test as applied to SH-SY5Y cultures [65]; 2 mM sodium azide has been shown directly to lower mitochondrial membrane potential and viability in SH-SY5Y [43]; and the bioenergetic consequences of Complex I-directed agents have been characterised in this line by extracellular flux analysis [52]. Published measurements in the same background are not condition-matched measurements in these cells, and we do not present them as such; they establish only that the exposures used here sit within the concentration range at which these compounds are known to engage mitochondrial function in SH-SY5Y. We also note that membrane-potential readouts under ATP-synthase blockade require care in interpretation, because the probes commonly used report a combination of plasma-membrane and mitochondrial potentials [66], resulting in a further reason that we prefer to state the absence of this measurement as a limitation (Section 3.11) rather than to infer it.

### 4.3. Incubation and Preparation of Cell Extracts

After complex-specific inhibition, the culture medium was aspirated and cells were washed once with PBS. Cells were then incubated in serum-free, glucose-free DMEM containing penicillin (100 units/mL), streptomycin (100 µg/mL), non-essential amino acids, and 5 mM [U-^13^C_6_]glucose, at 37 °C in a humidified atmosphere of 5% CO_2_/95% air for 24 h. At the end of the incubation, samples of culture medium were collected to analyse extracellular metabolites and assess glucose uptake. Cells were harvested and washed twice with cold PBS, and intracellular metabolites were extracted with 12% ice-cold perchloric acid. Cells from six plates were pooled for each analysis, neutralised with 5 M KOH, centrifuged at 15,000× *g* to remove insoluble KClO_4_ and lyophilised before storage at −80 °C until ^13^C NMR analysis. Specifically, the labelling medium was Gibco glucose-free DMEM (catalogue 11966) supplemented with 5 mM D-[U-^13^C_6_]glucose, 1% (*v*/*v*) MEM non-essential amino acids, penicillin (100 U/mL) and streptomycin (100 µg/mL); no foetal bovine serum and no sodium pyruvate were added. The base DMEM formulation contained 4 mM L-glutamine (unlabelled). This unlabelled glutamine contributes a constant background of mitochondrial carbon across all conditions and is one source of dilution of glutamate positional enrichment (Section 3.1). At harvest, cells were trypsinised, counted by trypan-blue exclusion, and pooled across six 100 mm plates per condition to give a matched viable-cell number per extract. Perchloric-acid extracts were neutralised and adjusted to a constant supernatant volume before lyophilisation, and lyophilised material was reconstituted in 300 µL D_2_O containing TSP (final 0.5 mM) as the chemical-shift and integration reference. ^13^C-NMR integrals were normalised to the TSP resonance, and the resulting values are reported as percent of within-batch control (same harvest day, matched cell number and matched extraction batch). Trypan-blue exclusion was used only as the operational anchor for inhibitor dose titration and does not provide independent information on metabolic state, apoptosis or ATP content. The use of [U-^13^C_6_]glucose tracing to infer pathway-associated carbon redistribution at steady state follows the framework summarised by Antoniewicz [15] and applications of [U-^13^C]glucose to neuronal PC/PDH compartmentation [13,14,16,17].

### 4.4. ^13^C NMR Acquisition

Lyophilised samples were dissolved in 300 µL of D_2_O. ^13^C NMR spectra were acquired at 201.2 MHz and 25 °C on an 800 MHz Bruker Avance spectrometer (Nuclear Magnetic Resonance Core Facility, Rensselaer Polytechnic Institute, Troy, NY, USA) equipped with a 5 mm ultra-sensitive HCN inverse triple-resonance cryoprobe. Acquisition parameters for the one-dimensional ^13^C spectra were as follows: 64k time-domain points, 2.3 s interpulse delay, 0.73 s acquisition time, 44.6 kHz spectral width, Waltz-16 ^1^H decoupling [67] and 2048 acquisitions. Spectral deconvolution was performed in MestReC (Mestrelab Research SL, Santiago de Compostela, Spain). Inversion-recovery T1 measurements were not performed on the present extracts. Representative literature ranges for ^13^C T1 of small protonated metabolites in aqueous solution at high field span approximately 0.5–2 s for protonated CH, CH_2_ and CH_3_ carbons and substantially longer (typically > 5 s) for quaternary carbons; under the 2.3 s interpulse delay used here, methylene and methine carbons are sampled close to full relaxation, while methyl carbons are partially saturated. The internal consistency of relative comparisons across conditions does not depend on absolute T1 values, because all comparisons are expressed as percent of within-batch control for the same resonance at identical acquisition parameters (same pulse program, relaxation delay, scan count, probe tuning and sample temperature), so any partial T1 saturation, NOE asymmetry or natural-abundance offset affects treated and control spectra equivalently and cancels in the ratio [15]. Quaternary carbons (long T1) are not used quantitatively and are excluded from Table 2. All ^13^C NMR experiments were performed in triplicate. Three independent biological replicates per condition were used (n = 3); each replicate represents a separate cell-culture, treatment and harvest cycle on a different day, with cells from six 100 mm plates pooled per replicate per condition. Technical NMR replicates on the same extract were not used to inflate n.

Resonance assignment and one correction to the primary data annotation. Assignments in Table 2 were made by chemical shift against the aqueous-solution values for the metabolites concerned and were checked for internal consistency against the shift ordering of the deconvolved spectrum. Two of them differ from the annotation carried in the primary integral tables, and both corrections are recorded here so that the archived data and the manuscript can be reconciled. First, the resonance at approximately 24.5 ppm was originally annotated GABA C2 under a numbering convention that counts from the amine-bearing carbon; under IUPAC numbering, used throughout this manuscript, that carbon is GABA C3, which is the position derived from glutamate C3 and therefore the PC-associated label. Second, the resonance at approximately 34.6 ppm, lying between glutamate C4 at 34.2 ppm and taurine C2 at 36.2 ppm, was originally annotated as a second GABA position under the same non-IUPAC convention; it is assigned here to succinate C2/C3. Three independent considerations support that assignment. By chemical shift, 34.6 ppm is the succinate C2/C3 position, and the resonance occupies the expected place in the shift ordering of the spectrum. By pattern of occurrence, the signal is absent from all control extracts and from MPTP and azide extracts, present in all three 3-NP replicates at roughly thirty times the normalised intensity seen under antimycin A or oligomycin, which is the distribution expected of succinate under covalent succinate dehydrogenase inactivation and not of a glutamate-derived GABA carbon. By internal consistency, the alternative assignment would require a large de novo appearance of a PDH-derived GABA carbon in the one condition in which glutamate C4 fell to 57% of control and glutamine C4 was undetected, which the rest of the dataset does not support. The assignment is also concordant with the ^13^C-NMR observation of Bakken and colleagues in cultured astrocytes, where ^13^C-labelled succinate was undetectable under control conditions and rose dose-dependently with 3-NP, while labelled aspartate was seen only in untreated cells [20]. The archived integral tables have been corrected to carry the IUPAC annotation used here, and the correspondence between the original and corrected labels is given in Supplementary Table S2 so that the reassignment is traceable.

### 4.5. Quantification of Metabolites by NMR

For lactate C3 and alanine C3, ^13^C fractional enrichments were calculated from the ^1^H spectra using the standard ratio of [^1^H–^12^C] singlet to flanking [^1^H–^13^C] satellite-doublet intensities at the methyl resonance; this ratio is self-normalising for ^13^C T1, NOE and natural-abundance contributions within a single spectrum [15]. Natural-abundance ^13^C contributions were corrected against parallel non-labelled control spectra. For all other metabolites in ^13^C, integrals were quantified by direct integration of the assigned resonance in the proton-decoupled ^13^C spectrum after deconvolution normalised to TSP (internal standard), and expressed as percent of the corresponding within-batch control integral. This representation is valid for comparing the same carbon position across treatments under matched acquisition conditions and is not converted to absolute concentration; systematic effects of partial T1 saturation, NOE asymmetry and natural-abundance contribution affect treated and control samples equivalently, and cancel in the percent-of-control ratio. Cross-carbon comparisons within a single spectrum are not attempted. Glutamate C4 and C3 positional enrichments are assigned and interpreted as PDH-associated (C4) and PC-associated (C3) labels following the well-established carbon-routing logic for [U-^13^C_6_]glucose entering the TCA cycle through the PDH/acetyl-CoA route versus the PC/oxaloacetate route [13,14,16,17]. Positional labels are pathway-associated readouts and not direct flux measurements [15]. No correction for isotopic scrambling was applied, and none can be derived from a single uniformly labelled glucose tracer sampled at one steady-state endpoint. Label entering through acetyl-CoA is redistributed between the C2/C3 and C4/C5 positions by passage through the symmetric intermediates succinate and fumarate, and positional asymmetry is expected to persist only through the first two to three turns of the cycle even where orientation-conserved transfer occurs between the succinate thiokinase, succinate dehydrogenase and fumarase steps [19]. At the 24 h endpoint used here, that limit was passed, so scrambling is assumed to be substantially complete, and the C4-versus-C3 contrast is treated as an integrated indicator of the relative weight of the two entry routes rather than as a route-specific measurement. This qualification is stated in the Results (Section 2.2), as well as in the note to Table 2.

### 4.6. Pyruvate Carboxylase Activity

PC catalyses pyruvate carboxylation to oxaloacetate using ATP and bicarbonate; the oxaloacetate generated is consumed by citrate synthase, and the free CoA released reacts with DTNB and was monitored at 412 nm and 30 °C [68]. Two reaction mixtures were prepared in separate UV-translucent cuvettes and kept on ice. Mixture 1 (control) contained 530 µL deionised water, 90 µL 1 M TrisHCl, 100 µL 0.5 M NaHCO_3_, 50 µL 0.1 M MgCl_2_, 100 µL 1 mM acetyl-CoA, 25 µL DTNB (3.9 mg in 1 mL ethanol), 5 µL citrate synthase (1000 U/mL) and 50 µL 0.1 M ATP. Mixture 2 contained the same components plus 50 µL 0.1 M pyruvate (final volume adjusted with water). Cell extract (50 µL) was added to Mixture 2, and rates were recorded spectrophotometrically. Activities are expressed relative to control. The assay is run on cell extracts in the presence of saturating exogenous MgATP, bicarbonate and acetyl-CoA, the last of which is the obligate allosteric activator of the enzyme and acts principally at the biotin-carboxylation domain where ATP cleavage occurs [47]. The readout is therefore the carboxylating capacity present in the extract under optimal cofactor supply, and not the rate at which the enzyme operated inside the intact cell, where MgATP and acetyl-CoA availability are themselves altered by the treatments.

### 4.7. Pyruvate Dehydrogenase Activity

PDH catalyses conversion of pyruvate to acetyl-CoA and CO2 using NAD^+^ as a co-substrate; PDH-derived acetyl-CoA was coupled to citrate synthase in the presence of oxaloacetate, and the released CoA was titrated with DTNB at 412 nm [69]. Mixture 1 (control) contained 325 µL deionised water, 200 µL 0.25 M TrisHCl, 50 µL 0.2 M sodium pyruvate, 50 µL 40 mM NAD^+^, 50 µL 40 mM thiamine pyrophosphate, 100 µL 10 mM MgCl_2_ and 50 µL 200 mM DTT. Mixture 2 contained the same components plus 50 µL 4 mM sodium-CoA (final volume adjusted with water). Cell extract (100 µL) was added to Mixture 2. To both mixtures, 50 µL oxaloacetate and 25 µL DTNB were added. Samples were equilibrated at 37 °C, and rates were recorded after addition of 5 µL citrate synthase. Activities are expressed relative to control. As for pyruvate carboxylase, exogenous NAD+, thiamine pyrophosphate and CoA are supplied in excess, so the readout reports the decarboxylating capacity of the extract rather than in-cell flux. Both enzyme readouts are therefore orthogonal to the labelling data in the sense that they are independent measurements made in parallel extracts prepared from the same biological replicates, but neither is a flux measurement, and the two should be read together rather than either alone. The perchloric-acid procedure described in Section 4.3 is a metabolite extraction that precipitates and denatures protein, and the material it yields cannot support an enzyme-activity assay; the PC and PDH assays were therefore performed on protein-preserving extracts prepared in parallel from the same biological replicates.

### 4.8. Statistical Analysis

All experiments were performed in three independent biological replicates per condition (n = 3), where each replicate represents a separate cell-culture, treatment and harvest cycle on a different day, with cells from six 100 mm plates pooled per replicate per condition. Technical replicates on the same extract were not used to inflate n. Data are expressed as mean +/− SD. Design and choice of test: The experiment is a single-factor, six-level design, meaning one vehicle-control group and five exposure groups, with three biological replicates each, and in which all five exposures are compared against the same control triplicate rather than against five separately prepared controls. The analysis was chosen to match that structure. ^13^C integrals, which are ratio-scale and bounded below by zero, were log2-transformed before analysis; this is the standard variance-stabilising transformation for relative metabolite data and removes the dependence of the within-group spread on the group mean that is otherwise characteristic of such measurements. For each metabolite, a one-way analysis of variance across all six groups was computed first, and the five exposure-versus-control contrasts were then obtained by Dunnett’s many-to-one procedure [70], which is the procedure defined for exactly this comparison structure and which controls the family-wise error rate across the five comparisons made against the shared control. Because Dunnett’s procedure pools the residual variance across all six groups, the error degrees of freedom are 12 rather than the 4 available to an isolated two-sample comparison, so the variance estimate on which every contrast rests is derived from 18 observations, and not from 6. On the assessment of assumptions: We did not screen the data for normality or for homogeneity of variance before selecting a test, and we do not report such screening. At three observations per group a Shapiro–Wilk or Levene test has essentially no power to detect a departure from either assumption, so a non-significant screening result would carry no information. More importantly, conditioning the choice of the main analysis on a preliminary test applied to the same data is itself a documented source of error: it alters the conditional Type I error rate of whichever test is subsequently run, for parametric and non-parametric alternatives alike [71], and selecting between pooled- and separate-variance forms of the t-test on the basis of the observed sample variances neither preserves the nominal significance level nor protects power [72]. The unconditional choice made here, a pooled-variance analysis across a balanced six-group design with the error variance estimated from all 18 observations, is therefore stated as a design decision rather than defended by a pretest. The balanced design is relevant: unequal variances are substantially less damaging to a pooled analysis when group sizes are equal, which they are throughout [73]. On non-parametric alternatives: Replacing the parametric test with a rank-based pairwise alternative is not available at this sample size. For a two-sided Mann–Whitney comparison of 3 against 3 the smallest attainable *p*-value is 0.1, and an exact permutation test on the same two groups has the same floor, so no pairwise comparison in this study could reach any conventional significance threshold under either procedure, regardless of the size of the effect. A distribution-free test is nevertheless available at the level of the design as a whole, because permuting 18 samples across six groups generates a null distribution that is not similarly constrained. We therefore tested the multivariate structure of the labelling data by permutational multivariate analysis of variance on Euclidean distances between the log2 profiles [74], with 9999 permutations of the group labels, obtaining pseudo-F = 18.77 and *p* = 0.0001 (Section 2.1). This provides the formal between-group hypothesis test that the earlier version of this manuscript stated it lacked, and it makes no distributional assumption. Effect sizes and sensitivity: Because significance thresholds are a weak basis for inference at this sample size, every exposure-versus-control contrast is also reported as Hedges’ g with a 95% confidence interval (Supplementary Table S1), and the principal conclusions are stated in terms of effect magnitude and of concordance with the orthogonal enzyme assays rather than in terms of threshold-crossing. No a priori power analysis was performed before the experiment; the sample size follows the convention of comparative ^13^C-NMR tracer studies in cultured cells, where three independent biological replicates per condition is usual, and that is stated as a limitation rather than presented as a justification. We have deliberately not computed post hoc or observed power, which is a monotone function of the *p*-value already reported and therefore adds no information to it [61,62]. The appropriate retrospective calculation is a sensitivity analysis, which asks what effect the completed design could have detected: at n = 3 per group, alpha = 0.05 and 80% power, a two-sample contrast detects a standardised effect of d = 3.07 or larger, and under the Dunnett procedure with five comparisons against a shared control and 12 error degrees of freedom, power is approximately 0.75 at d = 3.0 and 0.89 at d = 3.5. This study is therefore sensitive only to large effects, which is what it reports: the oligomycin glutamate C3 increase corresponds to g = 17.8, and the azide glutamate C4 decrease to g = −8.3, both far above the detection limit, whereas the comparisons that fail to reach significance under this analysis, principally in the MPTP condition, correspond to effects at or below it. Readers should treat the non-significant comparisons as uninformative rather than as evidence of no effect. Multiplicity across metabolites. The Dunnett adjustment applies within each metabolite, across the five exposure-versus-control comparisons. It does not adjust across the 22 resonances analysed, of which the 14 primary ones appear in Table 1 and the full set in Supplementary Table S1. We report the per-metabolite omnibus ANOVA *p*-value alongside the contrasts so that the reader can see which metabolites show any between-group structure at all, and we note that two of them, GABA C3 (*p* = 0.064) and aspartate C3 (*p* = 0.227), do not; no conclusion in this manuscript rests on either. A further false-discovery-rate correction across metabolites was considered, but it was not applied for two reasons. The metabolites are not independent tests of separate hypotheses but correlated readouts of a single carbon-routing process, so an independence-based adjustment would be conservative in an uninterpretable way. More importantly, the inferential structure of the study is hierarchical rather than metabolite-wide: the prespecified mechanistic endpoints are the PDH-associated glutamate C4 readout, the PC-associated glutamate C3 readout, their relationship, and the PC and PDH capacity assays, and the conclusions rest on these together with the multivariate permutation test and the reported effect sizes. The remaining resonances are exploratory, are reported for completeness and are not presented as established effects. Readers who prefer a metabolite-wide error rate can derive it from the per-contrast *p*-values in Supplementary Table S1; we have not applied one, because doing so would change the apparent status of exploratory endpoints on which no conclusion depends. Handling of undetected values and the ordination: Where a metabolite was detected in treated extracts but not in the corresponding control, no ratio to control can be formed. For the succinate row, a baseline-noise value was substituted for the undetected control signal in an earlier version of this analysis in order to obtain a finite ratio; because both the resulting ratio and any *p*-value derived from it are determined by the substituted constant rather than by the measurement, neither is reported, and succinate is given as detected or not detected (Table 1, footnote a). No imputation of any kind is used anywhere in the present analysis. Principal component analysis was computed on complete cases only: the seven metabolites detected in all three replicates of all six groups (lactate C3, alanine C3, glutamate C3, glutamate C4, glycine C2, citrate C2/C4 and alanine C2), expressed as log2 ratios to the mean of the control triplicate and autoscaled, giving an 18 × 7 matrix. We note explicitly that the variance percentages reported in the previous version of this manuscript were obtained from a matrix that included metabolites with undetected values, and they are superseded by the complete-case figures given in Section 2.1. The PERMANOVA was computed on the same complete-case matrix. Software and reproducibility: Analyses were performed in Python 3.12 using SciPy 1.17 (one-way ANOVA, Dunnett’s test), scikit-learn 1.8.0 (principal component analysis, with autoscaling) and NumPy 2.4.4, with the PERMANOVA implemented directly as a permutation of group labels on Euclidean distances between log2 profiles (9999 permutations, fixed random seed). The per-metabolite integral table, the analysis script and the full output, including every effect size and confidence interval, are provided as Supplementary Data so that the analysis can be reproduced exactly.

## 5. Conclusions

Exposure to five compounds nominally directed at Complexes I–V leaves undifferentiated SH-SY5Y cells in post-exposure ^13^C-labelling states that differ qualitatively rather than along a single severity axis, and exposure group has a significant overall effect on the multivariate labelling matrix (PERMANOVA pseudo-F = 18.77, *p* = 0.0001). Within the limits of a 24 h isotopic labelling endpoint and of this cell model, positional ^13^C-NMR distinguishes five states. 3-NP exposure produced a labelling situation suggesting succinate accumulation and disruption of TCA metabolism. Antimycin A exposure produced the broadest suppression of labelling, a metabolic labelling pattern consistent with redox suppression. Sodium azide exposure produced the strongest reduction in the PDH-associated glutamate C4 labelling signal (31.9% of control, *p* < 0.001, Hedges’ g = −8.3). MPTP exposure produced a comparatively weak and partial phenotype, with most individual comparisons not surviving the shared-control analysis. Oligomycin exposure produced the most distinct positional pattern, preserving the PDH-associated glutamate C4 readout (107.2%, *p* = 0.971) while nearly doubling the PC-associated glutamate C3 readout (197.7%, *p* = 0.004, g = 17.8); PC and PDH enzyme-capacity measurements in parallel extracts were directionally concordant with that pattern, providing orthogonal support for a stronger PC-associated positional-labelling signature under Complex V blockade. To our knowledge, this pairing of a positional ^13^C-NMR readout with PC and PDH capacity assays has not previously been reported for ATP synthase blockade in a neuronal-like cell, and we are not aware of a prior comparison of all five respiratory nodes by positional ^13^C-NMR in a single neuronal system. Because neither the NAD+/NADH ratio nor reactive oxygen species were measured, the antimycin A and MPTP readings are stated as labelling patterns consistent with a redox interpretation rather than as demonstrations of a redox mechanism. The experiment does not establish direct complex-specific flux: the tracer was supplied after washout, none of the compounds is fully selective for its nominal target, the enzyme assays report capacity and not rate, and no orthogonal pharmacological or genetic perturbation was applied at any node. The phenotypes reported here are therefore compound-defined post-exposure metabolic states. Where the corresponding bioenergetic endpoints have already been measured for these compounds in SH-SY5Y and related neuronal models, their direction is concordant with the present labelling data [41,42,43,44,52,65,75,76,77], and the pyruvate carboxylase and pyruvate dehydrogenase capacities measured here (Figure 4C,D) provide an orthogonal internal cross-check. Mechanistic attribution to individual respiratory complexes will require orthogonal tracers such as [U-^13^C_5_]glutamine, position-specific glucose tracers or ^13^C-bicarbonate at shorter time courses; condition-matched respirometry with ATP, membrane-potential, NAD+/NADH and ROS endpoints; 1H NMR of the adenylate pool to establish whether energy charge is persistently altered; chemically distinct comparators or genetic models at each node; and repetition in a differentiated or iPSC-derived neuronal model. Within those limits, the study identifies distinct post-exposure ^13^C-labelling phenotypes and supplies specific, testable hypotheses for follow-up flux work.

## Figures and Tables

**Table 1:** Intracellular ^13^C-labelled metabolites under complex-specific ETC inhibition. Values are mean +/− SD (n = 3), expressed as percent of the shared vehicle control. Positional labels are pathway-associated readouts, rather than direct flux measurements, and are subject to the isotopic scrambling stated in Section 2.2. n.d., not detected; an em dash indicates that no significance test could be computed. *P*-values are Dunnett many-to-one contrasts against the shared control, computed on log2-transformed values with the error variance pooled across all six groups (ANOVA df_error = 12); they are adjusted for the five comparisons made against that control within each metabolite and are given to three decimal places. The control group is shown as 100% by definition; its own replicate dispersion is not negligible and is reported here as the coefficient of variation of the control triplicate for each row: lactate C3, 29.2%; alanine C3, 9.6%; GABA C3, 48.5%; proline C4, 5.9%; Gln C3, 9.2%; GLT C3, 5.3%; Glu C3, 1.6%; proline C3, 13.4%; Gln C4, 33.0%; GLT C4, 31.8%; Glu C4, 10.3%; citrate C2/C4, 14.5%; and glycine C2, 6.2%. Table 1 lists the fourteen resonances that constitute the primary readout set. The full analysed set of twenty-two resonances, including alanine C2, aspartate C2, aspartate C3, taurine C1, taurine C2, glutamine C2, GLT C2 and glutamate C2, which are referred to in the Results and Discussion as exploratory endpoints, is given in Supplementary Table S1 together with Hedges’ g and 95% confidence intervals for every comparison reported here. The “a” means that succinate was not detected in control extracts, so no ratio to control and no significance test can be computed for that row, and none is reported. Succinate was detected in all three 3-NP replicates, at roughly thirty times the normalised intensity seen under antimycin A or oligomycin, and was absent under MPTP and azide. Values previously expressed relative to control for this row were obtained by substituting a baseline-noise value for the undetected control signal (Section 4.8) and are not reported here. The “b” indicates that Gln C3 (PC) was detected in three replicates of one exposure group only, so no many-to-one adjustment is defined; the value given is an unadjusted two-sample contrast and should be read as descriptive. The 3-NP GABA C3 entry derives from a single replicate, the metabolite being undetected in the other two.

Metabolite	MPTP		3-NP		AntiA		Azide		Oligo	
	% Control	*p* (Dunnett)	% Control	*p* (Dunnett)	% Control	*p* (Dunnett)	% Control	*p* (Dunnett)	% Control	*p* (Dunnett)
Lactate C3	48.3 ± 10.7	0.008	82.7 ± 17.1	0.790	20.9 ± 2.9	<0.001	19.4 ± 2.7	<0.001	28.4 ± 9.5	<0.001
Alanine C3	73.6 ± 12.6	0.259	82.3 ± 35.1	0.448	34.5 ± 5.5	<0.001	58.7 ± 3.8	0.028	75.6 ± 7.0	0.349
GABA C3	186.5 ± 3.5	0.069	37.3 (n = 1)	—	n.d.	—	n.d.	—	91.1 ± 6.3	>0.999
Proline C4	72.9 ± 5.6	0.029	45.4 ± 2.1	<0.001	40.4 ± 7.5	<0.001	n.d.	—	49.4 ± 8.2	<0.001
Gln C3 (PC)	50.9 ± 11.3	0.004 b	70.5 (n = 1)	—	n.d.	—	n.d.	—	n.d.	—
GLT C3	73.0 ± 29.0	0.281	29.7 (n = 1)	—	41.0 ± 9.2	0.005	48.5 ± 10.0	0.019	180.2 ± 30.7	0.062
Glu C3 (PC)	61.5 ± 8.5	0.030	56.8 ± 15.2	0.010	40.2 ± 12.4	<0.001	34.5 ± 4.2	<0.001	197.7 ± 8.1	0.004
Proline C3	98.3 ± 14.3	>0.999	29.2 ± 20.4 (n = 2)	—	4.3 ± 0.7	<0.001	63.0 ± 13.3	0.013	42.2 ± 3.4	<0.001
Gln C4 (PDH)	22.4 ± 14.8	0.003	n.d.	—	11.4 ± 0.3	<0.001	15.0 ± 5.1	<0.001	41.4 ± 15.0	0.087
GLT C4	33.5 ± 5.4	<0.001	n.d.	—	24.9 ± 4.3	<0.001	n.d.	—	142.1 ± 12.5	0.129
Glu C4 (PDH)	82.1 ± 4.2	0.476	57.4 ± 18.5	0.004	55.3 ± 10.4	0.003	31.9 ± 3.7	<0.001	107.2 ± 5.0	0.971
Succinate	n.d.	—	detected 3/3	—a	trace 3/3	—	n.d.	—	trace 3/3	—
Citrate C2/C4	72.1 ± 5.0	0.497	37.6 ± 13.5	0.003	40.3 ± 1.6	0.007	32.2 ± 13.8	<0.001	87.0 ± 13.7	0.951
Glycine C2	100.2 ± 22.2	>0.999	90.6 ± 22.7	0.887	43.7 ± 3.2	<0.001	41.0 ± 11.1	<0.001	110.6 ± 4.6	0.939

**Table 2:** Assignment of the observed ^13^C resonances to the precursor route each reports. Chemical shifts are approximate values for aqueous solution, given to orient the reader rather than as measured referencing. Route assignments are first-turn relationships: carbon entering through pyruvate dehydrogenase labels glutamate C4 and C5, while carbon entering through pyruvate carboxylase labels glutamate C2 and C3 via oxaloacetate. After 24 h of labelling, the system approaches isotopic steady state, at which point recirculation through the symmetric intermediates succinate and fumarate scrambles label between these positions; positional asymmetry is expected to persist only through the first two to three turns of the cycle even where orientation-conserved transfer occurs at the succinate thiokinase and succinate dehydrogenase steps [19], and at the 24 h endpoint used here, scrambling is assumed to be substantially complete. No scrambling correction was applied, and none is derivable from this tracer design (Section 2.2 and Section 4.5). The assignments are therefore pathway-associated approximations rather than measurements of flux through a single route. Carbon numbering follows IUPAC throughout; two resonances carry annotations in the primary integral tables that differ from those used here, and both corrections, including the assignment of the 34.6 ppm resonance to succinate C2/C3, are documented in Section 4.4 and Supplementary Table S2. Positions in glutamine, glutathione, proline and GABA are given with the parent glutamate carbon from which each derives, since all four retain the glutamate skeleton. The “a” for GLT denotes the resonances annotated as glutathione in Figure 1; its route assignment follows the glutamate skeleton of the gamma-glutamyl moiety and is therefore unaffected by that identification.

Resonance	δ (ppm)	Precursor Route Reported	Parent Glutamate Carbon
Alanine C3	17.1	Glycolytic; pyruvate C3 via alanine aminotransferase	—
Lactate C3	20.8	Glycolytic; pyruvate C3 via lactate dehydrogenase	—
GABA C3	24.5	Anaplerotic, PC-associated	Glu C3
Proline C4	24.7	Oxidative, PDH-associated	Glu C4
Glutamine C3	27.1	Anaplerotic, PC-associated	Glu C3
GLT C3 a	~27.5	Anaplerotic, PC-associated	Glu C3
Glutamate C3	27.8	Anaplerotic, PC-associated; oxaloacetate-derived	—
Proline C3	30.1	Anaplerotic, PC-associated	Glu C3
Glutamine C4	31.8	Oxidative, PDH-associated	Glu C4
GLT C4 a	~32.0	Oxidative, PDH-associated	Glu C4
Glutamate C4	34.2	Oxidative, PDH-associated; acetyl-CoA-derived	—
Succinate C2/C3	34.6	Symmetric intermediate; both routes contribute and the two carbons are indistinguishable	—
Taurine C2	36.2	Not on the PDH/PC axis; cysteine-derived, reached from glycolytic serine	—
Aspartate C3	37.3	Oxaloacetate C3	—
Glycine C2	42.4	Glycolytic; serine-derived from 3-phosphoglycerate	—
Citrate C2/C4	46.3	Reported as a single resonance; C2 is oxaloacetate-derived and C4 acetyl-CoA-derived, so the signal is mixed	—
Taurine C1	48.4	Not on the PDH/PC axis; cysteine-derived, reached from glycolytic serine	—
Alanine C2	51.4	Glycolytic; pyruvate C2 via alanine aminotransferase	—
Aspartate C2	53.2	Oxaloacetate C2	—
Glutamine C2	55.2	Anaplerotic, PC-associated	Glu C2
Glutamate C2	55.6	Anaplerotic, PC-associated; oxaloacetate-derived	—
GLT C2 a	~56.0	Anaplerotic, PC-associated	Glu C2

**Table 3:** Extracellular ^13^C-labelled glucose (α/β anomeric resonances), lactate C3 and alanine C3 in conditioned medium at the end of the 24 h labelling period, expressed as percent change relative to the shared vehicle control. Values derive from the same biological replicates as the intracellular data (n = 3 per group) and are normalised to the TSP internal standard in each medium spectrum. Each exposure was compared with the shared vehicle control by two-tailed, two-sample Student’s *t*-test assuming equal variance; *, *p* ≤ 0.05; **, *p* ≤ 0.01. These comparisons are unadjusted for multiplicity and are reported as descriptive, consistent with the treatment of the extracellular data as supporting, rather than mechanistic, evidence (Section 2.4 and Section 4.8).

Metabolite	MPTP	3-NP	AntiA	Azide	Oligo
**Glucose α**	−52.4 *	111.6 *	−44.3 **	75.3 **	−38.7 *
**Glucose β**	−52.2 *	103.10 *	−46.1 *	73.8 *	−36.5 *
**Lactate C3**	−0.90	60.60 *	73.8 *	140.3 *	**−6.0**
**Alanine C3**	**−22.2**	**37.4**	**42.3**	95.5 *	224.3 *

**Table 4:** On- and off-target pharmacology of the compounds used, and their consequences for interpretation of the labelling data. Concentrations are those used in the present study. “Origin” distinguishes activities that do not require inhibition of the nominal target from those that arise as a consequence of it.

Compound (Concentration Used)	Nominal Target and Reported Potency	Reported Activity Beyond the Nominal Target	Origin of that Activity	Consequence for the Present Readouts
**MPTP (1 mM)**	Complex I, via the MAO-B product MPP+ [28,29]; MPP+ concentrated in the matrix by the membrane potential [25]	Conversion of MPTP to MPP+ is limited in undifferentiated SH-SY5Y because MAO-B expression is low [7]	On-target but incomplete	The Complex I lesion is partial; this is the weakest of the five conditions and is interpreted only as a partial NADH-oxidation block
**3-NP (5 mM)**	Succinate dehydrogenase; covalent adduct on catalytic Arg-297 of SDH-A after oxidation to 3-nitroacrylate [21,31]	Secondary excitotoxic and calcium-mediated effects are reported in intact brain tissue	Requires glutamatergic circuitry absent from a cell monolayer	Not applicable to the present model; persistence of the on-target lesion is supported internally by ^13^C-succinate accumulation (Section 2.4)
**Antimycin A (4 ug/mL, ~7.3 µM)**	Qi site of cytochrome bc1; Ki = 0.033 nM [22], so the concentration used exceeds the on-target Ki by roughly five orders of magnitude	(a) Direct binding to the BH3 groove of Bcl-2/Bcl-xL, competitive with a Bak BH3 peptide; the 2-methoxy derivative of antimycin A3 retains this activity while losing respiratory inhibition [53]. (b) Destabilisation of c-Myc through ROS-dependent GSK3 activation, Thr-58 phosphorylation and proteasomal degradation [54]. (c) Opening of Cx43 hemichannels under metabolic inhibition, accompanying ATP depletion [55]	(a) Target-independent. (b) and (c) downstream of the on-target lesion, through ROS and ATP depletion respectively	(a) cannot be excluded at 7.3 µM and is treated as a genuine alternative contributor (Section 3.8). (b) and (c) are consequences of Complex III inhibition and therefore belong to the phenotype being described rather than confounding it
**Sodium azide (2 mM)**	Cytochrome c oxidase; chronic exposure causes irreversible loss of activity through holoenzyme dissociation [44]	Azide binds the metal centres of many haem proteins and metalloenzymes, and also inhibits the hydrolytic but not the synthetic activity of the F-ATPase [56], so inhibition of enzymes other than cytochrome c oxidase cannot be excluded at 2 mM	Target-independent, but chemically non-selective rather than directed at a defined second target	A broad haem-protein effect predicts broad suppression; it does not predict the observed selectivity for PDH-associated glutamate C4 over other labels
**Oligomycin (1 ug/mL, ~1.27 µM)**	Fo c-ring of F1Fo-ATP synthase [45]; sub-micromolar potency, with near-complete inhibition of ATP synthesis in human mitoplasts at <=1.25 µM [43]	Direct inhibition of store-operated calcium channels, IC_50_ ~ 2 µM, roughly 50-fold above the concentration blocking mitochondrial ATP production, with a rank order across oligomycins A, B and C differing from that for mitochondrial inhibition and with block of ICRAC under voltage clamp [57]. Inhibition of the ABC transporter Pdr5p has also been reported, but that transporter is a yeast protein with limited sequence homology and inverted topology relative to mammalian P-glycoprotein [58], so its relevance to this model is uncertain. Oligomycin A triggers IRE1-dependent endoplasmic-reticulum stress signalling with XBP1 splicing, CHOP induction and DR5 upregulation [59]; Akt and mTORC2 changes and altered autophagic flux are also reported after oligomycin exposure	Store-operated calcium channel block is target-independent. The stress-signalling and autophagy changes arise downstream of ATP-synthase blockade as part of the integrated response to energetic stress	The concentration used lies below the reported SOC IC_50_ but within the range where partial block cannot be excluded. Because calcium activates pyruvate dehydrogenase phosphatase [60], partial SOC inhibition is an alternative contributor to the reduced PDH activity observed under oligomycin, and is stated as such in Section 3.8

**Table 5:** Comparison of experimental design across the isotope-tracing studies of respiratory inhibition discussed in this work.

Study	Model	Perturbation	Tracer	Inhibitor Present During Tracing?	Analytical Readout
**Present study**	Undifferentiated SH-SY5Y	MPTP, 3-NP, antimycin A, azide, oligomycin (Complexes I–V)	[U-^13^C_6_]glucose, 24 h	No; removed before tracing	Positional ^13^C NMR plus PC and PDH activity assays
**Zhang et al. [18]**	Primary human retinal pigment epithelium	Piericidin, antimycin A, oligomycin (Complexes I, III, V)	^13^C-glucose	Yes, concurrent	LC-MS targeted metabolomics
**Basu and Blair [11]**	SH-SY5Y	Rotenone (Complex I)	[U-^13^C_6_]glucose	Yes, concurrent	LC-MS acyl-CoA thioester analysis
**Lei et al. [12]**	Dopaminergic cells	Paraquat, rotenone, MPP+, 6-OHDA	^13^C-glucose	Yes, concurrent	MS-based flux analysis
**Suciu et al. [50]**	Human LUHMES neurons	Rotenone, MPP+, berberine (Complex I), at non-ATP-depleting exposures	Metabolomic profiling	Yes, concurrent	Untargeted and targeted metabolomics
**Lussey-Lepoutre et al. [32]; Cardaci et al. [32]**	SDH-deficient cell lines	Genetic loss of SDH (chronic)	^13^C tracers	Not applicable; constitutive lesion	MS isotopologue analysis

## Data Availability

The per-replicate ^13^C integral table underlying Table 1, Figure 2 and Supplementary Table S1, together with the analysis script that reproduces the ANOVA, Dunnett contrasts, effect sizes, principal component analysis and PERMANOVA reported here, are provided as Supplementary Data. Raw NMR free induction decays are available from the corresponding author on reasonable request. No other datasets were generated or analysed.

## Supplementary Materials

The following supporting information can be downloaded at https://www.mdpi.com/article/10.3390/ijms27209160/s1.

## Abbreviations

The following abbreviations are used in this manuscript:

3-NP	3-nitropropionic acid
CcO	Cytochrome c oxidase
DTNB	5,5′-dithiobis(2-nitrobenzoic acid)
ETC	Electron transport chain
FBS	Foetal bovine serum
GABA	gamma-aminobutyric acid
MAO-B	Monoamine oxidase B
MPTP	1-methyl-4-phenyl-1,2,3,6-tetrahydropyridine
MPP+	1-methyl-4-phenylpyridinium
NMR	Nuclear magnetic resonance
OAA	Oxaloacetate
OXPHOS	Oxidative phosphorylation
PC	Pyruvate carboxylase
PCA	Principal component analysis
PDH	Pyruvate dehydrogenase
SSADH	Succinic semialdehyde dehydrogenase
ROS	Reactive oxygen species
SDH	Succinate dehydrogenase
SIRM	Stable isotope-resolved metabolomics
TCA	Tricarboxylic acid
