# Supplementary material for: Distinct Post-Exposure Metabolic States Following Pharmacological Perturbation of Mitochondrial Respiratory Complexes I–V in SH-SY5Y Cells: A Positional 13C-NMR Study
Source: Int J Mol Sci. 2026 Oct 15;27(20):9160. doi: 10.3390/ijms27209160 (PMC13705396; doi:10.3390/ijms27209160)
Supplement: Supplementary file 1 [file ijms-27-09160-s001.zip › ijms-4557950-supplementary.pdf]

Table S1. Effect sizes for every exposure vs control contrast

Hedges' g with 95% confidence interval, computed on log2-transformed values against the shared vehicle control. Omnibus p is the one-way ANOVA across all six groups for that metabolite; adjusted p is the Dunnett many-to-one contrast. n.d., metabolite not detected in that group; an em dash indicates that no test could be computed. a Detected in three replicates of one exposure group only, so no many-to-one adjustment is defined; the value is an unadjusted two-sample contrast and is descriptive. Reference: minimum detectable effect for this design is d = 3.07 (n = 3 per group, alpha = 0.05, 80% power).

| Metabolite | Control CV (%) | Omnibus p | Exposure | % control | Adjusted p | Hedges' g [95% CI] |
|---|---|---|---|---|---|---|
| **Lactate C3** | 29.2 | <0.0001 | MPTP | 48.3 ± 10.7 | 0.0081 | -2.3 [-4.6, -0.0] |
| **Lactate C3** | 29.2 | <0.0001 | 3-NP | 82.7 ± 17.1 | 0.7905 | -0.6 [-2.2, 1.1] |
| **Lactate C3** | 29.2 | <0.0001 | AntiA | 20.9 ± 2.9 | <0.0001 | -5.7 [-9.9, -1.4] |
| **Lactate C3** | 29.2 | <0.0001 | Azide | 19.4 ± 2.7 | <0.0001 | -5.9 [-10.3, -1.5] |
| **Lactate C3** | 29.2 | <0.0001 | Oligo | 28.4 ± 9.5 | <0.0001 | -3.4 [-6.3, -0.6] |
| **Alanine C3** | 9.6 | 0.0006 | MPTP | 73.6 ± 12.6 | 0.2585 | -1.8 [-3.9, 0.2] |
| **Alanine C3** | 9.6 | 0.0006 | 3-NP | 82.3 ± 35.1 | 0.4475 | -0.7 [-2.3, 1.0] |
| **Alanine C3** | 9.6 | 0.0006 | AntiA | 34.5 ± 5.5 | 0.0002 | -6.4 [-11.1, -1.7] |
| **Alanine C3** | 9.6 | 0.0006 | Azide | 58.7 ± 3.8 | 0.0278 | -5.1 [-9.0, -1.2] |
| **Alanine C3** | 9.6 | 0.0006 | Oligo | 75.6 ± 7.0 | 0.3487 | -2.4 [-4.6, -0.1] |
| **GABA C3** | 48.5 | 0.0637 | MPTP | 186.5 ± 3.5 | 0.0692 | 1.4 [-0.5, 3.3] |
| **GABA C3** | 48.5 | 0.0637 | 3-NP | 37.3 (n=1) | — | — |
| **GABA C3** | 48.5 | 0.0637 | AntiA | n.d. | — | — |
| **GABA C3** | 48.5 | 0.0637 | Azide | n.d. | — | — |
| **GABA C3** | 48.5 | 0.0637 | Oligo | 91.1 ± 6.3 | 0.9996 | 0.0 [-1.6, 1.6] |
| **Proline C4** | 5.9 | <0.0001 | MPTP | 72.9 ± 5.6 | 0.0290 | -3.7 [-6.7, -0.7] |
| **Proline C4** | 5.9 | <0.0001 | 3-NP | 45.4 ± 2.1 | <0.0001 | -12.0 [-20.5, -3.5] |
| **Proline C4** | 5.9 | <0.0001 | AntiA | 40.4 ± 7.5 | <0.0001 | -5.3 [-9.3, -1.3] |
| **Proline C4** | 5.9 | <0.0001 | Azide | n.d. | — | — |
| **Proline C4** | 5.9 | <0.0001 | Oligo | 49.4 ± 8.2 | 0.0001 | -4.7 [-8.4, -1.1] |
| **Gln C3 (PC)** | 9.2 | — | MPTP | 50.9 ± 11.3 | 0.0094 a | -3.1 [-5.7, -0.4] |
| **Gln C3 (PC)** | 9.2 | — | 3-NP | 70.5 (n=1) | — | — |
| **Gln C3 (PC)** | 9.2 | — | AntiA | n.d. | — | — |
| **Gln C3 (PC)** | 9.2 | — | Azide | n.d. | — | — |
| **Gln C3 (PC)** | 9.2 | — | Oligo | n.d. | — | — |
| **GLT C3** | 5.3 | 0.0002 | MPTP | 73.1 ± 29.0 | 0.2813 | -0.9 [-2.6, 0.8] |
| **GLT C3** | 5.3 | 0.0002 | 3-NP | 29.7 (n=1) | — | — |
| **GLT C3** | 5.3 | 0.0002 | AntiA | 41.0 ± 9.2 | 0.0051 | -4.5 [-8.1, -1.0] |
| **GLT C3** | 5.3 | 0.0002 | Azide | 48.5 ± 10.0 | 0.0186 | -4.0 [-7.2, -0.8] |
| **GLT C3** | 5.3 | 0.0002 | Oligo | 180.2 ± 30.7 | 0.0626 | 3.5 [0.6, 6.4] |
| **Glu C3 (PC)** | 1.6 | <0.0001 | MPTP | 61.5 ± 8.5 | 0.0297 | -4.0 [-7.3, -0.8] |
| **Glu C3 (PC)** | 1.6 | <0.0001 | 3-NP | 56.8 ± 15.2 | 0.0098 | -2.5 [-4.9, -0.1] |
| **Glu C3 (PC)** | 1.6 | <0.0001 | AntiA | 40.2 ± 12.4 | 0.0002 | -3.3 [-6.0, -0.5] |
| **Glu C3 (PC)** | 1.6 | <0.0001 | Azide | 34.5 ± 4.2 | <0.0001 | -10.0 [-17.1, -2.9] |
| **Glu C3 (PC)** | 1.6 | <0.0001 | Oligo | 197.7 ± 8.1 | 0.0034 | 17.8 [5.3, 30.2] |
| **Proline C3** | 13.4 | <0.0001 | MPTP | 98.3 ± 14.3 | 0.9996 | -0.1 [-1.7, 1.5] |
| **Proline C3** | 13.4 | <0.0001 | 3-NP | 29.2 (n=2) | — | — |
| **Proline C3** | 13.4 | <0.0001 | AntiA | 4.3 ± 0.7 | <0.0001 | -16.1 [-27.3, -4.8] |
| **Proline C3** | 13.4 | <0.0001 | Azide | 63.0 ± 13.3 | 0.0132 | -2.2 [-4.4, 0.0] |
| **Proline C3** | 13.4 | <0.0001 | Oligo | 42.2 ± 3.4 | 0.0002 | -6.3 [-10.9, -1.6] |
| **Gln C4 (PDH)** | 33.0 | 0.0006 | MPTP | 22.4 ± 14.8 | 0.0031 | -2.3 [-4.6, -0.0] |
| **Gln C4 (PDH)** | 33.0 | 0.0006 | 3-NP | n.d. | — | — |
| **Gln C4 (PDH)** | 33.0 | 0.0006 | AntiA | 11.4 ± 0.3 | 0.0003 | -6.4 [-11.1, -1.7] |
| **Gln C4 (PDH)** | 33.0 | 0.0006 | Azide | 15.0 ± 5.1 | 0.0009 | -4.0 [-7.2, -0.8] |
| **Gln C4 (PDH)** | 33.0 | 0.0006 | Oligo | 41.4 ± 15.0 | 0.0871 | -2.0 [-4.1, 0.1] |
| **GLT C4** | 31.8 | <0.0001 | MPTP | 33.4 ± 5.4 | 0.0005 | -3.2 [-6.0, -0.5] |
| **GLT C4** | 31.8 | <0.0001 | 3-NP | n.d. | — | — |
| **GLT C4** | 31.8 | <0.0001 | AntiA | 24.9 ± 4.3 | 0.0002 | -4.1 [-7.3, -0.8] |
| **GLT C4** | 31.8 | <0.0001 | Azide | n.d. | — | — |
| **GLT C4** | 31.8 | <0.0001 | Oligo | 142.1 ± 12.5 | 0.1288 | 1.2 [-0.6, 3.1] |
| **Glu C4 (PDH)** | 10.3 | <0.0001 | MPTP | 82.0 ± 4.2 | 0.4755 | -1.9 [-3.9, 0.2] |
| **Glu C4 (PDH)** | 10.3 | <0.0001 | 3-NP | 57.4 ± 18.5 | 0.0036 | -2.1 [-4.2, 0.1] |
| **Glu C4 (PDH)** | 10.3 | <0.0001 | AntiA | 55.3 ± 10.4 | 0.0030 | -3.1 [-5.7, -0.4] |
| **Glu C4 (PDH)** | 10.3 | <0.0001 | Azide | 31.9 ± 3.7 | <0.0001 | -8.3 [-14.2, -2.3] |
| **Glu C4 (PDH)** | 10.3 | <0.0001 | Oligo | 107.2 ± 5.0 | 0.9713 | 0.7 [-1.0, 2.4] |
| **Succinate C2/C3** | n.d. | — | MPTP | n.d. | — | — |
| **Succinate C2/C3** | n.d. | — | 3-NP | detected 3/3 | — | — |
| **Succinate C2/C3** | n.d. | — | AntiA | detected 3/3 | — | — |
| **Succinate C2/C3** | n.d. | — | Azide | n.d. | — | — |
| **Succinate C2/C3** | n.d. | — | Oligo | detected 3/3 | — | — |
| **Taurine C2** | 7.6 | 0.0823 | MPTP | 67.3 ± 50.9 | 0.2754 | -0.9 [-2.6, 0.8] |

| | | | | | | |
|---|---|---|---|---|---|---|
| **Taurine C2** | 7.6 | 0.0823 | 3-NP | 67.1 (n=2) | — | — |
| **Taurine C2** | 7.6 | 0.0823 | AntiA | 50.6 ± 16.4 | 0.1647 | -2.5 [-4.9, -0.2] |
| **Taurine C2** | 7.6 | 0.0823 | Azide | n.d. | — | — |
| **Taurine C2** | 7.6 | 0.0823 | Oligo | 35.4 ± 6.6 | 0.0391 | -6.1 [-10.6, -1.6] |
| **Aspartate C3** | 38.0 | 0.2267 | MPTP | 89.8 ± 30.1 | 0.9990 | -0.2 [-1.8, 1.4] |
| **Aspartate C3** | 38.0 | 0.2267 | 3-NP | 49.1 ± 57.1 | 0.1289 | -1.0 [-2.8, 0.7] |
| **Aspartate C3** | 38.0 | 0.2267 | AntiA | 58.2 ± 9.7 | 0.7141 | -1.2 [-3.0, 0.6] |
| **Aspartate C3** | 38.0 | 0.2267 | Azide | n.d. | — | — |
| **Aspartate C3** | 38.0 | 0.2267 | Oligo | 52.7 ± 11.9 | 0.5732 | -1.3 [-3.2, 0.5] |
| **Glycine C2** | 6.2 | <0.0001 | MPTP | 100.2 ± 22.2 | 1.0000 | -0.1 [-1.7, 1.5] |
| **Glycine C2** | 6.2 | <0.0001 | 3-NP | 90.6 ± 22.7 | 0.8865 | -0.5 [-2.1, 1.2] |
| **Glycine C2** | 6.2 | <0.0001 | AntiA | 43.7 ± 3.2 | 0.0007 | -9.7 [-16.6, -2.8] |
| **Glycine C2** | 6.2 | <0.0001 | Azide | 41.0 ± 11.1 | 0.0003 | -3.9 [-7.0, -0.7] |
| **Glycine C2** | 6.2 | <0.0001 | Oligo | 110.6 ± 4.6 | 0.9394 | 1.5 [-0.4, 3.4] |
| **Citrate C2/C4** | 14.5 | 0.0005 | MPTP | 72.1 ± 5.0 | 0.4967 | -2.3 [-4.6, -0.0] |
| **Citrate C2/C4** | 14.5 | 0.0005 | 3-NP | 37.6 ± 13.5 | 0.0031 | -2.9 [-5.5, -0.3] |
| **Citrate C2/C4** | 14.5 | 0.0005 | AntiA | 40.3 ± 1.6 | 0.0068 | -6.9 [-11.9, -1.8] |
| **Citrate C2/C4** | 14.5 | 0.0005 | Azide | 32.2 ± 13.8 | 0.0007 | -2.6 [-4.9, -0.2] |
| **Citrate C2/C4** | 14.5 | 0.0005 | Oligo | 87.0 ± 13.7 | 0.9507 | -0.7 [-2.4, 0.9] |
| **Taurine C1** | 25.8 | 0.0026 | MPTP | 44.9 ± 19.5 | 0.0117 | -1.9 [-4.0, 0.2] |
| **Taurine C1** | 25.8 | 0.0026 | 3-NP | n.d. | — | — |
| **Taurine C1** | 25.8 | 0.0026 | AntiA | 35.8 ± 4.2 | 0.0037 | -4.0 [-7.2, -0.8] |
| **Taurine C1** | 25.8 | 0.0026 | Azide | 28.7 ± 3.0 | 0.0009 | -4.9 [-8.7, -1.2] |
| **Taurine C1** | 25.8 | 0.0026 | Oligo | 43.7 ± 12.1 | 0.0123 | -2.4 [-4.6, -0.1] |
| **Alanine C2** | 16.3 | <0.0001 | MPTP | 70.4 ± 2.7 | 0.0829 | -2.3 [-4.6, -0.1] |
| **Alanine C2** | 16.3 | <0.0001 | 3-NP | 67.6 ± 7.4 | 0.0469 | -2.2 [-4.4, 0.0] |
| **Alanine C2** | 16.3 | <0.0001 | AntiA | 34.3 ± 7.2 | <0.0001 | -4.6 [-8.1, -1.0] |
| **Alanine C2** | 16.3 | <0.0001 | Azide | 22.6 ± 3.0 | <0.0001 | -7.9 [-13.7, -2.2] |
| **Alanine C2** | 16.3 | <0.0001 | Oligo | 32.3 ± 6.9 | <0.0001 | -4.6 [-8.2, -1.0] |
| **Aspartate C2** | 7.4 | 0.0157 | MPTP | 49.0 ± 29.6 | 0.3682 | -1.2 [-3.0, 0.6] |
| **Aspartate C2** | 7.4 | 0.0157 | 3-NP | n.d. | — | — |
| **Aspartate C2** | 7.4 | 0.0157 | AntiA | 28.5 ± 21.2 | 0.0682 | -1.5 [-3.4, 0.4] |
| **Aspartate C2** | 7.4 | 0.0157 | Azide | n.d. | — | — |
| **Aspartate C2** | 7.4 | 0.0157 | Oligo | 7.8 ± 1.5 | 0.0072 | -14.5 [-24.7, -4.3] |
| **Glutamine C2** | 12.4 | <0.0001 | MPTP | 129.8 ± 10.5 | 0.4467 | 2.0 [-0.1, 4.1] |
| **Glutamine C2** | 12.4 | <0.0001 | 3-NP | 153.3 ± 52.9 | 0.1681 | 1.2 [-0.6, 2.9] |
| **Glutamine C2** | 12.4 | <0.0001 | AntiA | 44.9 ± 9.1 | 0.0043 | -3.8 [-6.8, -0.7] |
| **Glutamine C2** | 12.4 | <0.0001 | Azide | n.d. | — | — |
| **Glutamine C2** | 12.4 | <0.0001 | Oligo | 36.8 ± 9.3 | 0.0009 | -4.2 [-7.6, -0.9] |
| **GLT C2** | 24.3 | 0.0036 | MPTP | 54.2 ± 9.0 | 0.4014 | -2.4 [-4.7, -0.1] |
| **GLT C2** | 24.3 | 0.0036 | 3-NP | 99.8 ± 65.3 | 0.9912 | -0.2 [-1.8, 1.4] |
| **GLT C2** | 24.3 | 0.0036 | AntiA | 17.4 ± 11.1 | 0.0023 | -2.6 [-5.0, -0.2] |
| **GLT C2** | 24.3 | 0.0036 | Azide | n.d. | — | — |
| **GLT C2** | 24.3 | 0.0036 | Oligo | 85.8 ± 8.7 | 0.9890 | -0.6 [-2.3, 1.0] |
| **Glutamate C2** | 18.3 | 0.0035 | MPTP | 58.7 ± 7.4 | 0.1327 | -2.8 [-5.3, -0.3] |
| **Glutamate C2** | 18.3 | 0.0035 | 3-NP | 66.7 ± 31.1 | 0.2080 | -1.1 [-2.9, 0.7] |
| **Glutamate C2** | 18.3 | 0.0035 | AntiA | 29.2 ± 11.5 | 0.0008 | -3.5 [-6.3, -0.6] |
| **Glutamate C2** | 18.3 | 0.0035 | Azide | n.d. | — | — |
| **Glutamate C2** | 18.3 | 0.0035 | Oligo | 70.9 ± 10.5 | 0.4271 | -1.7 [-3.6, 0.3] |

Table S2. Correspondence between primary file annotation and manuscript assignment

The primary integral tables (13C_quantitation_.xls) annotate two resonances under a numbering convention that counts from the amine-bearing carbon of GABA. The manuscript uses IUPAC numbering throughout. Both corrections are justified in Section 4.4. The archived data file has been updated to carry the manuscript annotation; this table preserves the correspondence so that the reassignment is traceable.

| delta (ppm) | Annotation in primary file | Assignment in manuscript | Basis for the assignment |
|---|---|---|---|
| **~24.5** | GABA C2 | GABA C3 | Non-IUPAC numbering in the source file counts from the amine-bearing carbon. Under IUPAC numbering this carbon is GABA C3, the position derived from glutamate C3 and therefore the PC-associated label. Shift ordering places it between alanine C3 (17.1 ppm) and proline C4 (24.7 ppm), as observed. |
| **~34.6** | GABA C3 (or "GABA") | Succinate C2/C3 | Chemical shift: 34.6 ppm is the succinate C2/C3 position, and the signal occupies the expected place in the shift ordering between glutamate C4 (34.2 ppm) and taurine C2 (36.2 ppm). Occurrence: absent from all control, MPTP and azide extracts; present in 3/3 3-NP replicates at ~30x the normalised intensity seen under antimycin A or oligomycin, the distribution expected of succinate under covalent SDH inactivation. Internal consistency: the GABA reading would require a large de novo appearance of a PDH-derived carbon in the one condition where glutamate C4 fell to 57% of control and glutamine C4 was undetected. Independent corroboration: Bakken et al. [65] report by 13C NMR in cultured astrocytes that labelled succinate is undetectable in control and rises dose-dependently with 3-NP. |
| **~48.4** | Tau C3 | Taurine C1 | Taurine is a two-carbon aminosulfonate and has no C3. The two observed taurine resonances are C1 and C2. |
